# Supplementary material for: Nitrogen, phosphorus and potassium formula fertilization model of Lonicera japonica Thunb in hilly and gully Loess Plateau of China
Source: PLoS One. 2025 Jun 4;20(6):e0323828. doi: 10.1371/journal.pone.0323828 (PMC12136625; doi:10.1371/journal.pone.0323828)
Supplement: Attachment — Submitted filename: PONE-D-24-06358_reviewer.pdf [file pone.0323828.s001.pdf]

PLOS ONE

Study on Nitrogen, Phosphorus and Potassium Formula Fertilization Model of Lonicera japonica Thunb in hilly and gully Loess Plateau of China

--Manuscript Draft--

**Manuscript Number:**	PONE-D-24-06358
**Article Type:**	Research Article
**Full Title:**	Study on Nitrogen, Phosphorus and Potassium Formula Fertilization Model of Lonicera japonica Thunb in hilly and gully Loess Plateau of China
**Short Title:**	N, P and K Formula Fertilization Model of Lonicera japonica Thunb in Loess Plateau of China
**Corresponding Author:**	Xiaofeng Jiang<br>Gansu Academy of Agricultural Sciences<br>Lanzhou, CHINA
**Keywords:**	L. japonica; Frequency analysis method; Fertilizer effect function.
**Abstract:**	Formula fertilization is effective in increasing yield and economic profit in medicinal plants, but no study on performance on Lonicera japonica Thunb (L. japonica) has been conducted, which is an important medicinal plant. Thus, the aim of this st[illegible]is to establish models for higher yield of L. japonica by formula fertilization with diffe[illegible] ratios of nitrogen (N), phosphorus (P) and potassium (K) in main producing areas in hilly and gully Loess Plateau of China. A '3414' formula fertilization trial with different dose and ratios of N, P, and K used for L. japonica was investigated. The fertilizer model was established by calculating and comparing the yield and economic profit of the plant . The results showed the application of N, P and K content in soil improved soil nutrient effectively. And P was identified as the primary limiting factor for yield. The ratio of output to investment of N2P2K3 was lowered significantly.The fertilizer effect function was: Y = 296.66 - 0.81X1 + 11.53X2 + 4.05X3 - 0.051X12 - 0.60X22 - 0.28X32 + 0.13X1×X2 + 0.20X1×X3 - 0.029X2×X3. The recommended dose of N, P, and K fertilizer was 18.25-26.75 g, 6.27-8.73 g, and 10.04-13.96 g, respectively, per plant. By applying the fertilizer with formula from this research, the yield and economic benefit increased notably by 38.18% and 2.71 CNY. These contribute significantly to the cultivation and production of L. japonica with improved yield and profit in specific geographical conditions of cultivating area for economic plant.
**Order of Authors:**	Xiaofeng Jiang
	Jianguo Guo
	Yuan Chen
	Shangzhong Li
	Zhahui Shi
	Ping Xu
	Jiali Chen
	Qing Fang
	Yusheng Zhai
**Additional Information:**	
**Question**	**Response**
**Financial Disclosure**<br><br>Enter a financial disclosure statement that describes the sources of funding for the work included in this submission. Review the submission guidelines for detailed	Yes

Powered by Editorial Manager® and ProduXion Manager® from Aries Systems Corporation

requirements. View published research articles from *PLOS ONE* for specific examples.<br><br>This statement is required for submission and **will appear in the published article** if the submission is accepted. Please make sure it is accurate.<br><br>**Funded studies**<br>Enter a statement with the following details:<br>• Initials of the authors who received each award<br>• Grant numbers awarded to each author<br>• The full name of each funder<br>• URL of each funder website<br>• Did the sponsors or funders play any role in the study design, data collection and analysis, decision to publish, or preparation of the manuscript?<br><br>Did you receive funding for this work?	
Please add funding details.<br>as follow-up to "**Financial Disclosure**<br><br>Enter a financial disclosure statement that describes the sources of funding for the work included in this submission. Review the submission guidelines for detailed requirements. View published research articles from *PLOS ONE* for specific examples.<br><br>This statement is required for submission and **will appear in the published article** if the submission is accepted. Please make sure it is accurate.	National key research and development project and sub-project (2021YFD1100504，2021YFD1100504-1，2021YFD1100504-2). Agricultural Science and technology independent innovation special key research and development program of Gansu Academy of Agricultural Sciences(2021GARS11); Gansu Province Science and Technology Plan project: Livelihood Science and technology special - Topics on rural revitalization(22CX2NA007); National Natural Science Foundation of China (NSFC)(31460547)

**Funded studies**<br>Enter a statement with the following details:<br>• Initials of the authors who received each award<br>• Grant numbers awarded to each author<br>• The full name of each funder<br>• URL of each funder website<br>• Did the sponsors or funders play any role in the study design, data collection and analysis, decision to publish, or preparation of the manuscript?<br><br>Did you receive funding for this work?"	
Please select the country of your main research funder (please select carefully as in some cases this is used in fee calculation).<br>as follow-up to "**Financial Disclosure**<br><br>Enter a financial disclosure statement that describes the sources of funding for the work included in this submission. Review the submission guidelines for detailed requirements. View published research articles from *PLOS ONE* for specific examples.<br><br>This statement is required for submission and **will appear in the published article** if the submission is accepted. Please make sure it is accurate.<br><br>**Funded studies**<br>Enter a statement with the following details:<br>• Initials of the authors who received each award<br>• Grant numbers awarded to each author<br>• The full name of each funder<br>• URL of each funder website<br>• Did the sponsors or funders play any role in the study design, data collection and analysis, decision to publish, or preparation of the manuscript?<br><br>Did you receive funding for this work?"	CHINA - CN
**Competing Interests**	no

Use the instructions below to enter a competing interest statement for this submission. On behalf of all authors, disclose any competing interests that could be perceived to bias this work—acknowledging all financial support and any other relevant financial or non-financial competing interests.<br><br>This statement is required for submission and **will appear in the published article** if the submission is accepted. Please make sure it is accurate and that any funding sources listed in your Funding Information later in the submission form are also declared in your Financial Disclosure statement.<br><br>View published research articles from *PLOS ONE* for specific examples.<br><br>**NO authors have competing interests**<br><br>Enter: *The authors have declared that no competing interests exist.*<br><br>**Authors with competing interests**<br><br>Enter competing interest details beginning with this statement:<br><br>*I have read the journal's policy and the authors of this manuscript have the following competing interests: [insert competing interests here]*<br><br>* typeset	
**Ethics Statement**<br><br>Enter an ethics statement for this submission. This statement is required if the study involved:<br><br>• Human participants<br>• Human specimens or tissue<br>• Vertebrate animals or cephalopods<br>• Vertebrate embryos or tissues	no

• Field research<br><br>Write "N/A" if the submission does not require an ethics statement.<br><br>General guidance is provided below. Consult the submission guidelines for detailed instructions. **Make sure that all information entered here is included in the Methods section of the manuscript.**	

Powered by Editorial Manager® and ProduXion Manager® from Aries Systems Corporation

**Format for specific study types**<br><br>**Human Subject Research (involving human participants and/or tissue)**<br>• Give the name of the institutional review board or ethics committee that approved the study<br>• Include the approval number and/or a statement indicating approval of this research<br>• Indicate the form of consent obtained (written/oral) or the reason that consent was not obtained (e.g. the data were analyzed anonymously)<br><br>**Animal Research (involving vertebrate animals, embryos or tissues)**<br>• Provide the name of the Institutional Animal Care and Use Committee (IACUC) or other relevant ethics board that reviewed the study protocol, and indicate whether they approved this research or granted a formal waiver of ethical approval<br>• Include an approval number if one was obtained<br>• If the study involved *non-human primates*, add *additional details* about animal welfare and steps taken to ameliorate suffering<br>• If anesthesia, euthanasia, or any kind of animal sacrifice is part of the study, include briefly which substances and/or methods were applied<br><br>**Field Research**<br><br>Include the following details if this study involves the collection of plant, animal, or other materials from a natural setting:<br>• Field permit number<br>• Name of the institution or relevant body that granted permission	
**Data Availability**<br><br>Authors are required to make all data underlying the findings described fully available, without restriction, and from the time of publication. PLOS allows rare exceptions to address legal and ethical concerns. See the PLOS Data Policy and FAQ for detailed information.	Yes - all data are fully available without restriction

A Data Availability Statement describing where the data can be found is required at submission. Your answers to this question constitute the Data Availability Statement and **will be published in the article**, if accepted.<br><br>**Important:** Stating 'data available on request from the author' is not sufficient. If your data are only available upon request, select 'No' for the first question and explain your exceptional situation in the text box.<br><br>Do the authors confirm that all data underlying the findings described in their manuscript are fully available without restriction?	
**Describe where the data may be found in full sentences. If you are copying our sample text, replace any instances of XXX with the appropriate details.**<br><br>• If the data are **held or will be held in a public repository**, include URLs, accession numbers or DOIs. If this information will only be available after acceptance, indicate this by ticking the box below. For example: *All XXX files are available from the XXX database (accession number(s) XXX, XXX.).*<br>• If the data are all contained **within the manuscript and/or Supporting Information files**, enter the following: *All relevant data are within the manuscript and its Supporting Information files.*<br>• If neither of these applies but you are able to provide **details of access elsewhere**, with or without limitations, please do so. For example:<br><br>*Data cannot be shared publicly because of [XXX]. Data are available from the XXX Institutional Data Access / Ethics Committee (contact via XXX) for researchers who meet the criteria for access to confidential data.*<br><br>*The data underlying the results presented in the study are available from (include the name of the third party*	This text is appropriate if the data are owned by a third party and authors do not have permission to share the data.

and contact information or URL).<br>• This text is appropriate if the data are owned by a third party and authors do not have permission to share the data.<br><br>* typeset	
Additional data availability information:	

Powered by Editorial Manager® and ProduXion Manager® from Aries Systems Corporation

Manuscript Click here to access/download;Manuscript;manuscript.docx

Study on Nitrogen, Phosphorus and Potassium Formula Fertilization Model of *Lonicera japonica Thunb* in hilly and gully Loess Plateau of China

Xiaofeng Jiang *[1,2,3,4], Jianguo Guo *[1], Yuan Chen[5], Shangzhong Li [1,2,3,4] , Zhahui Shi[6], Ping Xu[6], Jiali Chen [5], Qing Fang[5], Yusheng Zhai[6]

[1] Dryland Agriculture Institute / Institute of Plant Protection, Gansu Academy of Agricultural Sciences, Gansu Lanzhou,730070, China;

[2] Key Laboratory of High Water Utilization on Drvland of Gansu Province, Gansu Lanzhou,730070, China;

[3] Key Laboratory of Low-carbon Green Agriculture in Northwestern China,Ministry of Agriculture and Rural Affairs, Lanzhou, Gansu 730070, China;

[4] The Jiont Key Laboratory of Agriculture and Rural Affairs-Gansu Province for Crop Drought Resistance, Yield Increment and Rainwater Efficient Utilization on Rain-fed Area, Lanzhou, Gansu 730070, China;

[5] Agronomy College, Gansu Agricultural University, Gansu Lanzhou, 730070;

[6] Tongwei Qingliang Yuan Limited Liability Company of Lonicerae Japonicae Flos Industry Poverty Alleviation and Development,Gansu Dingxi 743300

[*] Corresponding Author:

Xiaofeng Jiang and Jianguo Guo; Dryland Agriculture Institute / Institute of Plant Protection, Gansu Academy of Agricultural Sciences, Gansu Lanzhou,730070, China.

Email addresses: jxf_5188@163.com; jguo1001@163.com

Abstract

Formula fertilization is effective in increasing yield and economic profit in medicinal plants, but no study on performance on *Lonicera japonica Thunb* (*L. japonica*) has been conducted, which is an important medicinal plant. Thus, the aim of this study is to establish models for higher yield of *L. japonica* by formula fertilization with different ratios of nitrogen (N), phosphorus (P) and potassium (K) in main producing areas in hilly and gully Loess Plateau of China. A '3414' formula fertilization trial with different dose and ratios of N, P, and K used for *L. japonica* was investigated. The fertilizer model was established by calculating and comparing the yield and economic profit of the plant . The results showed the application of N, P and K content in soil improved soil nutrient effectively. And P was identified as the primary limiting factor for yield. The ratio of output to investment of $N_2P_2K_3$ was lowered significantly.The fertilizer effect function was: $Y = 296.66 - 0.81X_1 + 11.53X_2 + 4.05X_3 - 0.051X_1^2 - 0.60X_2^2 - 0.28X_3^2 + 0.13X_1 \times X_2 + 0.20X_1 \times X_3 - 0.029X_2 \times X_3$. The recommended dose of N, P, and K fertilizer was 18.25-26.75 g, 6.27-8.73 g, and 10.04-13.96 g, respectively, per plant. By applying the fertilizer with formula from this research, the yield and economic benefit increased notably by 38.18% and 2.71 CNY. These contribute significantly to the cultivation and production of *L. japonica* with improved yield and profit in specific geographical conditions of cultivating area for economic plant.

Keywords: *L. japonica*; Frequency analysis method; Fertilizer effect function.

1. Introduction

Lonicera japonica Thunb (*L. japonica*), also known as honeysuckle, belongs to *Lonicera* genus in the Caprifoliaceae family [1]. Gansu Province is a main producing area in China. Their buds are harvested and dried before bloom in early summer, which are rich in organic acids, flavonoids, terpenoids, and volatile oils, so they have been widely recognized as effective medicinal herbs for heat-clearing and detoxification, and are commonly used in clinical practice for treating various

diseases including pneumonia [2]. Recently, the market demand for honeysuckle is increasing significantly due to the impact of COVID-19. As a result, artificial cultivation has become the primary method of obtaining raw materials [3]. However, research on efficient cultivation techniques for honeysuckle is still in its early stages. Therefore, high-quality and efficient production of *L. japonica* is in urgent need to overcome traditional improper fertilization practices.

Formula fertilization is an effective way to improve yield and quality of *L. japonica*. Former research found that combined application on N, P and K could not only promote the biosynthesis of Chlorogenic acid and flavonoids in *L. japonica*, but also increase the yield and quality [4-7]. For example, the single application of nitrogen fertilizer in Japan promoted the vegetative growth of *L. japonica*, increased the biological yield of vegetative organs and the crude protein content of leaves, and the economic yield and Chlorogenic acid content of the buds of reproductive organs were decreased [8-11]. Adding P could not only promote flower bud differentiation and increase economic yield, but also increase the Chlorogenic acid content of flower buds and improve commodity quality [12-14]. When the recommended dosages of N, P_2O_5 and K_2O were 34.9 - 47.9, 13.1 - 14.4, 18.5 -23.8 g/plant, the economic yield of *L. japonica* was 387.0 - 430.3 CNY/plant [15, 16].

Tongwei County locates in the hilly and gully region of the Loess Plateau in central Gansu Province. The landform in the territory is composed of hilly and gully like hills, hilly and gully, residual tableland and Sichuan tableland. The mountain ridges are undulating, and the ravines are crisscrossing. The annual Sunshine duration are 1400 h, and the annual total solar radiation is 5446.5 $MJ.m^{-2}$. There are abundant light and heat resources, which are conducive to the development of modern Silk Road agricultural practices in cold and arid areas. Currently, a high-end *L. japonica* planting base spanning 6,667 ha has been established within the Tongwei Qingliang Yuan Limited Liability Company. This endeavor is part of the Poverty Alleviation and Development initiative in the *L. japonica* Industry. Moreover, the produced *L.*

japonica has achieved a remarkable status, both in terms of quality and industry reputation, being at the forefront within China. However, due to long-term planting, there has been a significant increase in fertilizer usage for per tree, which has become a major hindrance to the development of local industries. There is an urgent need to investigate the optimal dosage of N, P, and K fertilizers for *L. japonica*. Therefore, this study conducted an experiment using "3414" Fertilizer Design experiment to establish a model and finally determine a optimal formula for cultivating *L. japonica* with high yield and economic profit.

2. Materials and Methods

2.1 Overview of the test site

The test began in April 2022. The test site located in Wangjiahe Community, Yaochuan Village, Lidian Township, Tongwei County (1860 m above sea level, 35 ° 25 ′ 40 ″ N, 105 ° 10 ′ 30 ″ E). It belongs to temperate semi-arid Continental climate type, with four distinct seasons, drought and little rain, and abundant light resources. The average annual temperature was 8.2℃, and the annual precipitation was 300mm, Most of them are from July to September. The frost free period was 145 d. The crops mature once a year and belong to the hilly and gully agricultural area of the Loess Plateau in central Gansu. The development of *L.japonica* industry has unique natural advantages.The soil pH of the experimental site was 8.49. The organic matter in soil was 11.01 g/kg. total N was 0.6 g/kg, available N was 42.80 mg/kg. Total P was 0.72 g/kg, available P was 8.97 mg/kg, total K was 17.15 g/kg, and the available K was 160.67 mg/kg.

2.2 Materials

The tested *L. japonica* species was Beihua No.1 of 4 year's cultivating time. The fertilizers used in the test were mainly urea (total N content is 46%, Petro China Company Limited), granular Superphosphate (the effective P_2O_5 was 12%, Baoji Fenghe Chemical Co., Ltd.), Potassium sulfate (K_2O was 50%, Qinghai Laishuo Industry and Trade Co., Ltd.).

2.3 Method

2.3.1 Design of experiments

This experiment adopted quadratic regression optimal design scheme of ‘3414’ with 3 fertilizer factors set as N, P, and K, and the levels were 0, 1, 2, and 3 [10]. There were 14 treatments totally. Among them, of which level 0 means with no fertilization; level 2 means the commonly used fertilization amount; level 1 means 1/2 of the commonly used fertilization amount; and level 3 means 3/2 of the commonly used fertilization amount. This was the first time for formula fertilization experiment carried out on the Tongwei *L. japonica* in the hilly and gully area of the Loess Plateau in central Gansu. There is no reference data for the determination of the 2 levels of each factor (the approximate value of the local optimal fertilization amount). Therefore, it was determined by reference to the local yield level in 2016-2021 and the fertilization amount in Tongwei County. Each plant was applied with 30 g of pure N, 10 g of P_2O_5, and 16 g of K_2O. Each treatment had 6 *L. japonica* trees, 3 replicates, arranged by random blocks, and the spacing between plants and rows was 1.0 mX1.0 m. Nitrogen fertilizer, phosphorus fertilizer, and potassium fertilizer were applied in a one-time manner on April 8, 2022 before germination.The method of fertilization was ditch application, and the fertilization amount was determined according to Table 1.

Table 1 Experimental design of ‘3414’ test.

Number	Treatment	N-coded value	P-coded value	K-coded value	Fertilization amount (kg.hm^{-2})		
					N	P_2O_5	K_2O
1	$N_0P_0K_0$	0	0	0	0	0	0
2	$N_0P_2K_2$	0	2	2	0	10	16
3	$N_1P_2K_2$	1	2	2	15	10	16
4	$N_2P_0K_2$	2	0	2	30	0	16
5	$N_2P_1K_2$	2	1	2	30	5	16
6	$N_2P_2K_2$	2	2	2	30	10	16

7	$N_2P_3K_2$	2	3	2	30	15	16
8	$N_2P_2K_0$	2	2	0	30	10	0
9	$N_2P_2K_1$	2	2	1	30	10	8
10	$N_2P_2K_3$	2	2	3	30	10	24
11	$N_3P_2K_2$	3	2	2	45	10	16
12	$N_1P_1K_2$	1	1	2	15	5	16
13	$N_1P_2K_1$	1	2	1	15	10	8
14	$N_2P_1K_1$	2	1	1	30	5	8

2.3.2 Measurement items and methods

Yield measurement: the total yield was picked by hand and was calculated in June.

2.3.3 Data analysis

DPS7.5 software and Exce1 2010 software were used to fit the univariate quadratic, binary quadratic and ternary quadratic fertilizer effect functions of yield and fertilizer application, and whether they comply with typical fertilizer efficiency models was analyzed. If it belongs to an atypical fertilizer efficiency model, the frequency analysis method was used to calculate the economic optimal fertilizer amount and the maximum yield fertilizer amount.

The frequency analysis calculation formula was [10, 17]:

$$\overline{x_i} = \frac{1}{n_j}\sum_{j=0}^{p} u_j\, n_{ij} (i = 1,2,3) \qquad (1)$$

In the formula, n_{ij} represents the number of occurrences of the variable value X_{ij}, where (u_j), and n_j represents the total number of occurrences of the variable X_i.

Standard deviation:

$$S\overline{x_i} = \frac{1}{\sqrt{n_j-1}} \cdot \sqrt{\sum_{j=0}^{p}\left(u_j - \overline{x_i}\right)^{\cdot 2} n_{ij}} \qquad (2)$$

Mean standard deviation:

$$S_x = \frac{S}{\sqrt{n_j}} \qquad (3)$$

95% confidence interval：

$$\left(\overline{x_i} - S\overline{x_i} \cdot t_{0.05(n_j-1)}\right) \sim \left(\overline{x_i} + S\overline{x_i} \cdot t_{0.05(n_j-1)}\right) \qquad (4)$$

3. Result

3.1 Effects of Different Fertilization Treatments on the Yield and Economic Benefits of *L. japonica*

The index of soil nutrient abundance and deficiency was expressed as the percentage of yield in the nutrient deficient area to the total yield in fertilizer deficient area. That is, the relative yield can express the abundance and deficiency of soil nutrients. Soil nutrients with a relative yield below 50% are extremely low; 50% -75% is low; 75% -95% is medium; greater than 95% is high. Thus, the index of soil nutrient abundance and deficiency of a certain crop in a certain area and the corresponding fertilizer amount were determined [15-16]. (It is high to determine the soil nutrient abundance and deficiency index of a certain crop in a certain area and the corresponding fertilization amount.). From Table 2, it can be seen that the contribution rate of basic soil fertility in the field trial site is 73.97%. The yield in the non fertilized area accounts for 73.97% of the total yield in the fully fertilized area, the yield in the N deficient area accounts for 84.67% of the total yield in the fully fertilized area, the yield in the P deficient area accounts for 78.70% of the total yield in the fully fertilized area, and the yield in the K deficient area accounts for 80.72% of the total yield in the fully fertilized area. The results showed that the content of available N, P and K in soil was on medium grade, the soil's ability to provide nitrogen, phosphorus, and potassium is on an average degrade. Therefore, the P content of soil is the primary fertility factor that limits the yield of *L. japonica*. Within the recommended fertilization range, each application with 1 g of N, P, and K fertilizer can increase the yield of buds by 2.04 g, 8.52 g, and 4.82 g, respectively. The results showed that increasing P fertilizer had a significant effect on improving yield of *L. japonica* buds.

Table 2 The relative yield and increasing rate of fertilizer deficiency treatments

Processing number	Yield ($g.plant^{-1}$)	Relative yield (%)	Increased yield of 1g fertilizer production of bud fresh weight（g）		
			N	P_2O_5	K_2O

1	$N_0P_0K_0$	295.77	73.97	-	-	-
2	$N_0P_2K_2$	338.53	84.67	2.04	-	-
4	$N_2P_0K_2$	314.67	78.70	-	8.52	-
8	$N_2P_2K_0$	322.73	80.72	-	-	4.82
6	$N_2P_2K_2$	399.83	100.00	-	-	-

From Table 3, it can be seen that the yield of *L. japonica* per plant was greatly affected by different treatments. Among them, the yield and economic benefits of $N_2P_3K_2$ treatment were the highest at 409 g per plant and 9.81 CNY per plant. The treatment of $N_2P_3K_2$ increased the yield significantly by 38.18% compared to the control, while the yield and economic benefits of the non fertilized control were the lowest on 296 g per plant and 7.10 CNY per plant. The highest output to investment ratio was the treatment of $N_1P_2K_1$, which reached 47.26. However, the lowest output ratio for output to investment ratio was $N_2P_2K_3$, which was 23.96. For treatments of $N_0P_2K_2$, $N_1P_2K_2$, $N_2P_2K_2$, $N_3P_2K_2$, and $N_2P_0K_2$, $N_2P_1K_2$, $N_2P_2K_2$, and $N_2P_3K_2$ in Table 3, it can be seen that N and P fertilizers can promote the increase of yield per plant ,and the yield per plant of *L. japonica* gradually increases with the increase of fertilizer amount. From the treatments of $N_2P_2K_2$, $N_2P_2K_0$, $N_2P_2K_1$, and $N_2P_2K_3$ in From Table 2, it can be seen that as rate of P increases in production, the yield increased at low ratios and reached its highest value at treatment of No.6. However, when the amount of P application continued to increase until to treatment 10, the yield of per plant did not increase any more, but decreased slightly. The results of interaction relationship of fertilizer was PK>NP>NK, and the yield increased 14.46%, 9.12% and 6.39%, respectively compared with treatment with no fertilizer.

Table 3 Effects of different treatments on *L. japonica* yield and economic benefits

Experiment number		Yield (g.plant^{-1})	Increased profit than control (%)	Economic profit (CNY/ plant)	Fertilizer cost (CNY/ plant)	Ratio of profit to investment
1	$N_0P_0K_0$	295.77 ± 2.85j	-	7.10	0.000	-
2	$N_0P_2K_2$	338.5 ± 2.21h	14.46	8.12	0.188	43.22
3	$N_1P_2K_2$	389.7 ± 5.30c	31.76	9.35	0.260	35.97
4	$N_2P_0K_2$	314.67 ± 9.08i	6.39	7.55	0.272	27.76
5	$N_2P_1K_2$	378.5 ± 10.41de	27.97	9.08	0.302	30.08
6	$N_2P_2K_2$	399.83 ± 8.13ab	35.19	9.60	0.332	28.90
7	$N_2P_3K_2$	408.70 ± 2.86a	38.18	9.81	0.362	27.10
8	$N_2P_2K_0$	322.73 ± 1.16i	9.12	7.75	0.204	37.97
9	$N_2P_2K_1$	372.27 ± 4.92ef	25.86	8.93	0.268	33.34
10	$N_2P_2K_3$	395.27 ± 1.68bc	33.64	9.49	0.396	23.96
11	$N_3P_2K_2$	407.93 ± 4.14a	37.92	9.79	0.404	24.23
12	$N_1P_1K_2$	367.80 ± 4.78f	24.35	8.83	0.230	38.38
13	$N_1P_2K_1$	386.73 ± 5.14cd	30.76	9.28	0.196	47.36
14	$N_2P_1K_1$	353.4 ± 10.83g	19.49	8.48	0.238	35.64

Note: The '-' indicates does not exist, the unit price of *L. japonica* is 24 CNY. kg^{-1}, N is 4.8 CNY. kg^{-1}, P_2O_5 is 6 CNY. kg^{-1}, and $K_2O$is 8 CNY. kg$^{-1.}$

3.2Analysis of fertilizer effect function

3.2.1 Analysis of Ternary Quadratic Fertilizer Effect Function

Using DPS software to perform regression analysis between independent variables x and y (yield per plant), the fertilizer effect function was calculated as: $Y=296.66-0.81X_1+11.53X_2+4.05X_3-0.051X_1^2-0.60X_2^2-0.28X_3^2+0.13X_1 * X_2+0.20X_1 * X_3-0.029X_2 * X_3$; where 0.81, 11.53 and 4.05 are the absolute values of the first term of the equation, respectively. The results showed that the yield of *L. japonica* was affected by the single factor of N, P, and K in the order of P > K > N. Indicating that it can be seen that P fertilizer had the greatest impact on the yield of honeysuckle,

while N fertilizer had the smallest effect. This result was consistent with results in Table 2.

The correlation coefficient R^2 was 0.9938 (P=0.0018<0.05) obtained by using DPS software for multiple regression analysis and test. It passed F-test and reached a significant level. It showed that there was a high correlation between independent variables x and y (yield). and there was a significant regression relationship between yield of *L. japonica* and N, P, K fertilizers, but the coefficient of the first term in the equation had both positive and negative values, which did not conform to the typical ternary quadratic fertilizer effect function. The calculation of recommended fertilization amount by using the marginal derivative method was not consistent with actual production. Therefore, frequency analysis method is used to calculate the recommended fertilization amount.

Analysis of single factor fertilizer effect function Single factor refers to fixing the fertilizer level of two factors and the relationship equation between fertilizer change and yield change was fitted. From Table 2, it can be seen that the fitting of the single factor fertilizer effect function was the same as that of the 3 factors, and the fertilizer usage and yield of N, P, and K fertilizers were applied for fitting. Regression analysis was conducted on N fertilizer effect function and correlation coefficients with F and P values using treatments N numbered 2, 3, 6, and 11 in Table 1. Similarly, the effect equations of P fertilizer were fitted using treatments 4, 5, 6, and 7, while the effect equations of K fertilizer were fitted using treatments 6, 8, 9, and 10. The effect equations of each fertilizer are detailed in Table 2. The results showed that the correlation coefficient R of the fertilizer effect function of N fertilizer was 0.9868 (P=0.1621>0.05), and the regression effect of the equation was not significant. The correlation coefficient R of the fertilizer effect function of P fertilizer was 0.9955, (P=0.0944>0.05), and the regression effect of the equation is not significant. The correlation coefficient R of the fertilizer effect function of K fertilizer is 0.9990 (P=0.0440<0.05), and the regression effect of the equation is significant.

From Table 5, it can be seen that the maximum fertilization amounts of N, P_2O_5, and K_2O for each factor in the single factor fertilizer effect function are 7.5, 13, and 19.29, respectively. The corresponding maximum fertilization amounts are 408.45 g/ plant, 409.03 g/plant, and 445.99 g/plant, respectively.

Table 4 Analysis on single factor fertilizer effect function

Factor	Fertilizer effect function	R^2	P
N fertilizer	$Y=340.95+3.60X_1-0.048X_1^2$	0.9868	0.1621
P fertilizer	$Y=316.55+14.31X_2-0.55X_2^2$	0.9955	0.0944
K fertilizer	$Y=322.40+8.11X_3 -0.21X_3^2$	0.9990	0.0440

Table 5 Single factor fertilizer effect function each factor fertilizer application and maximum yield analysis

Factor functions	Maximum fertilization amount of each factor（$g.plant^{-1}$）	Maximum output（$g.plant^{-1}$）
Fertilizer effect function of N	7.5	408.45
Fertilizer effect function of P	13	409.03
Fertilizer effect function of K	19.29	445.99

3.2.2 Analysis of Two-Factor Fertilizer Effect Function

The two-factor fertilizer equation was an equation that fixes level of one fertilizer unchanged and fits the changes in the levels of two fertilizers with the changes in yield. The fitting of the interaction effect function between the two factors was the same as that of the three factors. From Table 6, it can be seen that the regression analysis was performed on the fertilizer dosage and yield through NP, NK, and PK fertilizer related treatments. The interaction effect equation of NP fertilizer was obtained through regression analysis between the fertilizer dosage and yield of treatments numbered 2, 3, 4, 5, 6, 7, 11, and 12 in Table 1. Similarly, the interaction effect equation of NK fertilizer was obtained through regression analysis of fertilizer dosage 2 and yield under treatments of 2, 3, 6, 8, 9, 10, 11, and 13. The interaction effect equation of PK fertilizer was obtained through regression analysis of fertilizer dosage 2 and yield under treatments of 4, 5, 6, 7, 8, 9, 10, and 14. The results showed

that the correlation coefficient R^2 of the fertilizer effect function of NP fertilizer was 0.9946, $P=0.0269<0.05$, indicating a significant regression effect of the equation; The correlation coefficient R^2 of the fertilizer effect function of NK fertilizer was 0.9898, $P=0.0498<0.05$, and the regression effect of the equation was significant; The correlation coefficient R^2 of the fertilizer effect function of PK fertilizer was 0.9948, $P=0.0259<0.05$, and the equation regression effect was significant.

Table 6 Analysis on fertilizer effect function of two factors

Factor	Fertilizer effect function	R^2	P
NP fertilizer	$Y=303.10+1.94X_1+9.72X_2-0.051X_1^2-0.60X_2^2+0.18X_1*X_2$	0.9946	0.0269
NK fertilizer	$Y=382.48-0.80X_1+1.35X_3-0.042X_1^2-0.24X_3^2+0.25X_1*X_3$	0.9898	0.0498
PK fertilizer	$Y=260.41+11.11X_2+7.36X_3-0.50X_2^2-0.24X_3^2+0.15X_2*X_3$	0.9948	0.0259

3.2.3 Production frequency analysis

According to the above analysis, the regression effects of N and P fertilizers were not significant in the single factor fertilizer effect function, while the regression effects of K fertilizers were significant. The regression effects of NP, NK, and PK fertilizers were significant in the fertilizer effect functions of the interaction of two factors. The ternary quadratic fertilizer effect function was not a typical ternary quadratic fertilizer effect function. Therefore, this study was not suitable for using the marginal derivative method to calculate the maximum yield fertilization amount and the optimal fertilization amount. For results that did not fit the typical ternary quadratic fertilizer effect function, the yield frequency analysis method could be used to determine the appropriate recommended fertilization amount.

3.2.4 Setting Statistical Intervals (Yield Range)

According to the results of the field experiment (Table 7), the yield range was set. The maximum actual average yield of this experimental community was 409 g/plant, and the minimum was 296 g/plant. The yield range (statistical interval) was set to $296<y<409$. Frequency analysis was calculated based on the field yield statistical table of *L. japonica*.

Table 7 Field trial yield of *L. japonica*

Number	Treatment	Nutrient consumption (g.plant $^{-1}$)			Yield per plant (g.plant $^{-1}$)			
		N	P_2O_5	K_2O	I	II	III	Average value
1	$N_0P_0K_0$	0	0	0	297	296	295	296
2	$N_0P_2K_2$	0	10	16	337	338	340	339
3	$N_1P_2K_2$	15	10	16	386	396	387	390
4	$N_2P_0K_2$	30	0	16	313	307	325	315
5	$N_2P_1K_2$	30	5	16	376	370	390	379
6	$N_2P_2K_2$	30	10	16	391	402	407	400
7	$N_2P_3K_2$	30	15	16	406	412	408	409
8	$N_2P_2K_0$	30	10	0	324	323	322	323
9	$N_2P_2K_1$	30	10	8	370	369	378	372
10	$N_2P_2K_3$	30	10	24	395	397	394	395
11	$N_3P_2K_2$	45	10	16	407	413	405	408
12	$N_1P_1K_2$	15	5	16	364	373	366	368
13	$N_1P_2K_1$	15	10	8	390	381	390	387
14	$N_2P_1K_1$	30	5	8	359	341	360	353

3.3 Yield frequency analysis

The frequency of each factor level of nitrogen, phosphorus and potassium falling into the set yield range was counted respectively, and the average, standard deviation, standard deviation, and 95% confidence interval recommended fertilization amount of nitrogen, phosphorus, and potassium according to formulas (1) - (4) were calculated to obtain the yield frequency analysis of honeysuckle, as shown in Table 8. By calculation, The Times of nitrogen fertilizer yield falling into the set interval at 0-3 level were 5, 9, 21 and 2 times, The Times of phosphate fertilizer yield falling into the set interval at 0-3 level were 5,9,24 and 3 times, and The Times of potassium fertilizer

yield falling into the set interval at 0-3 level were 5,9,24 and 3 times, respectively. The average fertilization rates of nitrogen, phosphorus, and potassium fertilizers were 23.57,7.86 and 12.57, respectively. The recommended fertilization amount for nitrogen fertilizer (N) is 18.25-26.75 g/plant, for phosphorus fertilizer (P_2O_5) is 6.27-8.73 g/plant, and for potassium fertilizer (K_2O) is 10.04-13.96 g/plant, resulting in a yield of 376.45-397.94 g/plant.

Table 8 Frequency analysis of *L. japonica* output

	N fertilization amount			P fertilization amount			K fertilization amount		
Fertilization level	Fertilization amount ($g.plant^{-1}$)	Frequency	Rate(%)	Fertilization amount ($g.plant^{-1}$)	Frequency	Rate (%)	Fertilization amount ($g.plant^{-1}$)	Frequency	Rate (%)
0	0	5	13.51	0	5	12.20	0	5	12.20
1	15	9	24.32	5	9	21.95	8	9	21.95
2	30	21	56.76	10	24	58.54	16	24	58.54
3	45	2	5.41	15	3	7.32	24	3	7.32
Sum of times		37			41			41	
x_i average value	22.5			7.5			12		
Standard deviation (s)	13.17			4.05			6.40		
Standard deviation	2.17			0.63			1.00		
95%confidence interval	18.25-26.75			6.27-8.73			10.04-13.96		

3.4 Calculation and Optimization of Fertilization Rate and Yield

The average application rates of nitrogen, phosphorus, and potassium fertilizers obtained by frequency analysis was 22.5 g/plant, 7.5 g/plant, and 12 g/plant, which were brought into the ternary quadratic fertilizer effect function, and the optimized fertilization yield was calculated to be 386.95 g/plant.

4. Discussion

Inorganic elements such as N, P and K are the mineral elements with the largest demand for plant metabolism, tissue and organ formation, and plant growth and development. Fertilization was used in agricultural production to meet needs of plants for these three elements [18]. Cao *et al* [19] had shown that balanced fertilization of N,

P, and K can significantly promote plant growth and development (such as the number of flower nodes, fresh weight of flowers, and number of flower buds), and improve flower bud yield. Research had shown that fertilization could help improve the yield of *L. japonica*. As the amount of fertilization increased, the yield per plant also increased. However, when the amount of fertilization reached a certain level, the yield immediately decrease [20, 21]. Through a single experiment on N, P, and K, it was found that the order of effect on increasing yield of *L. japonica* is: N>P>K. Among them, the appropriate application rates were 0.1, 0.2, and 0.1 $kg.plant^{-1}$, respectively [22]. The results of yield study showed that N, P, K, and compound fertilizers could promote the differentiation of flower buds in honeysuckle plants, which were positively correlated with the length of flower buds. Among them, N could increase the yield of leaves and flowers, while P was the main fertility factor affecting flower bud weight and yield, and was the key element in flower bud growth and development [23]. In this study, different fertilizer ratios had a significant impact on the yield of *L. japonica* per plant; compared to the control, the yield of treatment $N_2P_3K_2$ had increased by 38.18%. The contribution rate of basic soil fertility in the field trial site was 73.97%. Within the recommended fertilization amount range, applying 1 g of nitrogen, phosphorus, and potassium fertilizer could increase the yield of *L. japonica* buds by 2.04, 8.52 and 4.82 g, respectively. The results indicated that P fertilizer was the main limiting factor for improving the yield of *L. japonica* buds, and the amount of phosphorus fertilizer should be paid attention to in order to achieve high yield, which was consistent with the results of Zeng Huijie et al [18]. In Tongwei County in the hilly and gully region of the Loess Plateau in central Gansu, the correlation coefficient R^2 of the equation function of *L. japonica* fertilizer effect established by regression analysis based on the yield of *L. japonica* obtained by experiment and the amount of N, P and K was 0.9938, $P=0.0018<0.05$, indicating a significant regression effect. However, the coefficient of the first term were -0.81, 11.53 and 4.05 respectively, which were a typical fertilizer effect functions, and did not conform to

the law of Diminishing returns. The F-test *P*=0.0018<0.05 reached a significant level, and the marginal analysis method was used to solve the optimal fertilization amount and the maximum yield fertilization amount, which was out of line with the actual production. Therefore, the recommended fertilization amount of 4-year-old *L. japonica* per plant calculated by frequency analysis method was nitrogen fertilizer(N), which was 18.25-26.75g.plant^{-1}. The recommended fertilization amount for phosphorus fertilizer (P_2O_5) was 6.27-8.73 g/plant, while for potassium fertilizer (K_2O), the recommended fertilization amount was 10.04-13.96 g/plant, and the yield per plant was 376.45-397.94 g/plant. However, the conventional fertilization amount determined by referring to the local yield level and fertilization amount in Tongwei County from 2016 to 2021was 30 g/plant of pure nitrogen, 10 g/plant of P_2O_5, and 16 g/plant of K_2O 16, which was higher than the recommended fertilization amount calculated by frequency analysis. Therefore, the recommended fertilization amount for the next year's test should be adjusted slightly lower.

To sum up, due to the differences in climate conditions, soil texture and fertility, fertilization should be designed and adjusted according to the specific conditions of each planting region. At the same time, attention should be paid to balanced use of inorganic fertilizer and Manure to avoid soil hardening and imbalance of microbial flora, so as to better promote the development of *L. japonica* industry.

5. Conclusion

This study demonstrated that formula fertilization with different ratios of N, P, and K can significantly increase the yield of fresh buds of *L. japonica* in cultivating area with poor soil nutrient in China. The application of formula fertilizer with N, P, and K improved soil nutrient to a medium grade, with P content being the primary limiting factor for the yield. Specifically, increasing the dose of N, P, and K fertilizers at the recommended levels resulted in a significant increase in the yield. As a result, the yield of per plant reached 409 g, with an economic benefit of 9.81 CNY g/plant. This represents a substantial increase of 38.18% in yield and a significant economic benefit

of 2.71 CNY g/plant compared to the control. To model this effect, a function was derived as: $Y=296.66-0.81X_1+11.53X_2+4.05X_3-0.051X_1^2-0.60X_2^2-0.28X_3^2+0.13X_1 \times X_2+0.20X_1 \times X_3-0.029X_2 \times X_3$. Taking into account the combined application of N, P_2O_5, and K_2O fertilizers per plant. Through frequency analysis, the recommended doses of N, P_2O_5, and K_2O fertilizers were determined to be within the ranges of 18.25~26.75 g/plant, 6.27~8.73 g/plant, and 10.04~13.96 g/plant, respectively. The findings of this study have practical implications for the cultivation and production of *L. japonica* and provide valuable insights for optimizing cultivation practices and maximizing the potential of this important medicinal plant in the specific geographical conditions of cultivating area.

Funding

National key research and development project and sub-project (2021YFD1100504, 2021YFD1100504-1, 2021YFD1100504-2). Agricultural Science and technology independent innovation special key research and development program of Gansu Academy of Agricultural Sciences(2021GARS11); Gansu Province Science and Technology Plan project: Livelihood Science and technology special - Topics on rural revitalization(22CX2NA007); National Natural Science Foundation of China (NSFC)(31460547)

Conflicts of Interest

The authors have no conflicts of interest to declare.

References：

1. Liu Z, Cheng Y, Chao Z. A Comprehensive Quality Analysis of Different Colors of Medicinal and Edible Honeysuckle. Foods. 2023;12(16):3126.
2. Xiao O, Li M, Chen D, Chen J, Simal-Gandara J, Dai X, et al. The dissipation, processing factors, metabolites, and risk assessment of pesticides in honeysuckle from field to table. Journal of Hazardous Materials. 2022;431:128519.
3. Qin S, Chen X, Jiang C, Li M, Yuan Y, Yang J, et al. Pruning induced yield and quality variations and the correlated gene expression and phytohormone changes in Lonicera japonica. Industrial Crops and Products. 2019;132:386-95.
4. Zhang X, Li S, An X, Song Z, Zhu Y, Tan Y, et al. Effects of nitrogen, phosphorus and potassium formula fertilization on the yield and berry quality of

blueberry. Plos one. 2023;18(3):e0283137.

5. Zhao F, Li F, Zhou J, Sun X, Wang Y, Jing L, et al. Soiltesting formula fertilization with organic fertilizer addition for target yield cannot stand long due to stem lodging of rice. Frontiers in Plant Science. 2022;13:1091156.
6. Bao Q-p, Han X-r, Cui Z-g, Yin Y, Zhang H, Li W-r, et al. Recommended fertilization model for spring maize nitrogen fertilizer in Northeast China. Journal of Plant Nutrition and Fertilizers. 2020;26(4):705-16.
7. Saquee FS, Diakite S, Kavhiza NJ, Pakina E, Zargar M. The Efficacy of Micronutrient Fertilizers on the Yield Formulation and Quality of Wheat Grains. Agronomy. 2023;13(2):566.
8. Guo L, Qiao J, Zhou D, Qin D, Huo J. Effect of Vermicompost on Fruit Quality, Growth, and Rhizosphere Soil Enzyme Activities of Blue Honeysuckle (Lonicera caerulea L.). Journal of Soil Science and Plant Nutrition. 2023:1-9.
9. Dyess JG, Causey MK, Stribling HL, Lockaby BG. Effects of fertilization on production and quality of Japanese honeysuckle. Southern Journal of Applied Forestry. 1994;18(2):68-71.
10. Chen Y, Zhou X, Ma L, Lin Y, Huang X. Chinese yam yield is affected by soil nutrient levels and interactions among N, P, and K fertilizers. Chinese Herbal Medicines. 2023.
11. Liao G, Wang Y, Yu H, He P, Lin Z, Dai T, et al. Nutrient use efficiency has decreased in southwest China since 2009 with increasing risk of nutrient excess. Communications Earth & Environment. 2023;4(1):388.
12. Iseki K, Ikazaki K, Batieno BJ. Heterogeneity effects of plant density and fertilizer application on cowpea grain yield in soil types with different physicochemical characteristics. Field Crops Research. 2023;292:108825.
13. Yang L, Lin Y, Kong J, Yu Y, He Q, Su Y, et al. Effects of fertilization and dry-season irrigation on the timber production and carbon storage in subtropical Eucalyptus plantations. Industrial Crops and Products. 2023;192:116143.
14. Tang S, Zhou J, Pan W, Sun T, Liu M, Tang R, et al. Effects of combined application of nitrogen, phosphorus, and potassium fertilizers on tea (Camellia sinensis) growth and fungal community. Applied Soil Ecology. 2023;181:104661.
15. Fontes PCR, Braun H, Busato C, Cecon PR. Economic optimum nitrogen fertilization rates and nitrogen fertilization rate effects on tuber characteristics of potato cultivars. Potato Research. 2010;53:167-79.
16. Li H, Zhang Y, Sun Y, Liu P, Zhang Q, Wang X, et al. Long-term effects of optimized fertilization, tillage and crop rotation on soil fertility, crop yield and economic profit on the Loess Plateau. European Journal of Agronomy. 2023;143:126731.
17. Wang Q, Wang X-c, Li X-y, Wang B, Gao X-q, Fu B-z. Effect of “3414” F[illegible]ization on Seed Yield and Quality of Bromus inermis in Arid Area of Ningxia. Acta Agrestia Sinica. 30(12):3470.

18. Zeng H, Wang X, Qiao Z, Li Y, Cai N, Liu S, et al. Influence on yield and quality of Lonicera japonica by soil testing and formulated fertilization. J Nucl Agric Sci. 2017;31:2443-9.
19. Cao Y, Pan Y, Wang M, Liu T, Meng X, Guo S. The effects of different nitrogen forms on chlorophyll fluorescence and photosystem II in Lonicera japonica. Journal of Plant Growth Regulation. 2023;42(7):4106-17.
20. Gardner Z. Effects of Fertilization and Drying Conditions on the Quality of Selected Chinese Medicinal Plants. 2019.
21. Chen K, Ma L, Chen C, Liu N, Wang B, Bao Y, et al. Long-Term Impact of N, P, K Fertilizers in Different Rates on Yield and Quality of Anisodus tanguticus (Maxinowicz) Pascher. Plants. 2023;12(11):2102.
22. Jourdan C, Silva EV, Gonçalves JLM, Ranger J, Moreira RM, Laclau JP. Fine root production and turnover in Brazilian Eucalyptus plantations under contrasting nitrogen fertilization regimes. Forest ecology and management. 2[illegible];256(3):396-404.
23. Zeng P, Guo Z, Xiao X, Peng C, Liu L, Yan D, et al. Physiological stress responses, mineral element uptake and phytoremediation potential of Morus alba L. in cadmium-contaminated soil. Ecotoxicology and environmental safety. 2020;189:109973.
