# Supplementary material for: Nitrogen, phosphorus and potassium formula fertilization model of Lonicera japonica Thunb in hilly and gully Loess Plateau of China
Source: PLoS One. 2025 Jun 4;20(6):e0323828. doi: 10.1371/journal.pone.0323828 (PMC12136625; doi:10.1371/journal.pone.0323828)
Supplement: Attachment — Submitted filename: PONE-D-24-06358_R4_reviewer.pdf [file pone.0323828.s010.pdf]

PLOS ONE

Study on Nitrogen, Phosphorus and Potassium Formula Fertilization Model of Lonicera japonicaThunb in hilly and gully Loess Plateau of China

--Manuscript Draft--

**Manuscript Number:**	PONE-D-24-06358R4
**Article Type:**	Research Article
**Full Title:**	Study on Nitrogen, Phosphorus and Potassium Formula Fertilization Model of Lonicera japonicaThunb in hilly and gully Loess Plateau of China
**Short Title:**	N, P and K Formula Fertilization Model of Lonicera japonica Thunb in Loess Plateau of China
**Corresponding Author:**	Xiaofeng Jiang<br>Gansu Academy of Agricultural Sciences<br>Lanzhou, CHINA
**Keywords:**	L. japonica; Frequency analysis method; Fertilizer effect function; 3414 experiment; Soil testing formula fertilization.
**Abstract:**	Formula fertilization is effective in increasing yield and economic profit in medicinal plants, Lonicera japonicaThunb (L. japonica) is an important medicinal plant, but no study on performance of formula fertilization has been conducted, yet. Thus, the aim of this study is to establish models for higher yield of L. japonica by formula fertilization with different ratios of nitrogen (N), phosphorus (P) and potassium (K) in main producing areas in hilly and gully Loess Plateau of China. A '3414' formula fertilization trial with different dose and ratios of N, P, and K used for L. japonica was investigated. The fertilizer was poured into a small pit of 30 cm deep around the root of each tree. The spacing between plants and rows was 1.0 m X 1.0 m. The fertilizer model was established by calculating and comparing yield and economic profit of the plant. The results showed application of N, P and K content in soil improved yield effectively. And P was identified as the primary limiting factor for yield. The ratio of output to investment of N2P2K3 was lowered significantly. The fertilizer effect function was: Y = 296.66 - 0.81X1 + 11.53X2 + 4.05X3 - 0.051X12 - 0.60X22 - 0.28X32 + 0.13X1×X2 + 0.20X1×X3 - 0.029X2×X3. The recommended dose of N, P, and K fertilizer was 18.25-26.75 g, 6.27-8.73 g, and 10.04-13.96 g per plant. By applying formula fertilizer from this study, the yield and economic benefit increased notably by 38.18% and 2.71 CNY per plant. These contribute significantly to production of L. japonica with improved yield and profit in specific geographical conditions of cultivating area for economic plant.
**Order of Authors:**	Xiaofeng Jiang
	Jianguo Guo
	Yuan Chen
	Shangzhong Li
	Zhahui Shi
	Ping Xu
	Jiali Chen
	Qing Fang
	Yusheng Zhai
**Response to Reviewers:**	Response to Reviewer Comments<br>Dear Editors and Reviewers:<br>We are very grateful for the further comments concerning our manuscript entitled "Study on Nitrogen, Phosphorus and Potassium Formula Fertilization Model of Lonicera japonica Thunb in hilly and gully Loess Plateau of China (PONE-D-24-06358)". These comments are all valuable and very helpful for the further revision and improvement of our article, which also provides an important guideline for our researches and scientific writing. We have carefully studied the comments and made corrections that we hope meet the standards for publication. If you have any questions

Powered by Editorial Manager® and ProduXion Manager® from Aries Systems Corporation

regarding this manuscript, please do not hesitate to contact me. The detailed corrections in the article and the responses to the editors' and reviewers' comments are as follows:

Response to Editor Requirements:
1.Line 40. Please Change CNY by dollars or euros? Frequently, all international journal use USA dollars.
Answer: Based on Exchange rate between US Dollar and RMB on December 12, 2024:1USD≈7.2674CNY 1CNY≈ 0.1376USD
The yuan in the original text is completely revised to US dollars.(Line40)
2.Line 47. Remove Lonicera genus in the
Answer:“belongs to Lonicera genus in the Caprifoliaceae family” instead“belongs to the Caprifoliaceae family”(Line46-47)
3.Line 100. Please put soil texture. This information is essential to exchange cationic capacity.
Answer: The soil is loam。(Line101)
4.Line 103 is it grams of active ingredient? If yes. It should be g ai.Please consider this sentence: Imidacloprid wettable (25%), powder with 1000 fold solution, were spray at rate of 24 g per hectare for the plant.
Answer: yes. “Imidacloprid wettable (25%), powder with 1000 fold solution, were spray at rate of 24 g per hectare for the plant”instead“ Imidacloprid 25% wettable powder were sprayed at rate of 24 g a.i. per hectare for the plant”(line103-104)
5.108. Please put space in L. japonica
Line 113. Why do the authors not mention K in the title? And line 198 mention K
Answer: The original has been amended.(line108)
The author mentions potassium in the title.
6.122. I think 3 fertilizers and 4 levels involve 40 treatments. Why the research was done with only 14 treatments.
N0Q0P0 ; N0Q0P1; N0Q1P1 ; N0Q1P2 ; N0Q2P2 ; N0Q2P3 ; N0Q3P3 ; N0Q1P3; N0Q2P3; N0Q3P2 ; N1Q0P0 ; N1Q0P1; N1Q1P1 ; N1Q1P2 ; N1Q2P2 ; N1Q2P3 ; N1Q3P3 ; N1Q1P3; N1Q2P3; N1Q3P2 ; N2Q0P0 ; N2Q0P1; N2Q1P1 ; N2Q1P2 ; N2Q2P2 ; N2Q2P3 ; N2Q3P3 ; N2Q1P3; N2Q2P3; N2Q3P2 ; N3Q0P0 ; N3Q0P1; N3Q1P1 ; N3Q1P2 ; N3Q2P2 ; N3Q2P3 ; N3Q3P3 ; N3Q1P3; N3Q2P3;N3Q3P2.
Answer: 3414 test design 14 processing is because it is a special design based on Latin square design, this design can effectively control the test error and improve the accuracy of the test.
7.Line 173 and 174. Under this title explain the amount of N, P and K o the soil as a result; however, there is not information in material of methods about how to do this research
Answer: Soil total nitrogen content was determined by NY/T 53-1987 standard Kjeldt nitrogen determination method, soil total phosphorus content was determined by NY/T 88-1988 standard molybdenum-antimony resistance colorimetric method, and soil total potassium content was determined by NY/T 87-1988 standard acid melting flame photometer and FP6450 type flame photometer.(line147-152)
8.Line 216 and 217 both cite that fertilizer increase the yield per plant
Answer: “it can be seen that N and P fertilizers can promote the increase of yield per plant ,and the yield per plant of L. japonica gradually increases with the increase of fertilizer amount. ”instead“With the increase of nitrogen and phosphate fertilizer amount, the yield per plant of L. japonica increased gradually. ”(line220-222)
9.The authors should check the references. For example: Number 23 is not cited in the text.
Answer:The original has been amended.(line357)
10.Line 319. Delete one standard deviation
Answer: Deleted from the original text.(line322)
11.Line from 340 to 349 is information according to introduction section no discussion section.

Powered by Editorial Manager® and ProduXion Manager® from Aries Systems Corporation

	Answer: Thank you for your valuable suggestions！ We have corrected the manuscript as you required. “In this study, the yield of L. japonica increased with the increase of nitrogen and phosphorus content.”(line357-359)<br>12.Line 339 please put L. Japonica in cursive in the discussion and conclusion section.<br>Answer: The original has been amended.(line368,373,388)<br>13.Line 388-390. The two sentence join by a comma has not sense together.<br>Answer: Thank you for your valuable suggestions！ We have corrected the manuscript as you required. “The results indicated that phosphorus (P) fertilizer was the primary limiting factor for enhancing the yield of L. japonica buds. Therefore, it is essential to consider the amount of phosphorus fertilizer applied to achieve high yields and a finding that aligns with the results of Zeng Huijie et al.”(line388-392)<br>14.Line 413: in summery…. has information that not was previously mentioned such as weather variation or texture.<br>Answer: Thank you for your valuable suggestions！ We have corrected the manuscript as you required. “In summary, due to soil texture, and fertility, fertilization strategies should be tailored to the specific conditions of each planting region. Additionally, it is essential to ensure a balanced application of inorganic fertilizers to prevent soil compaction and imbalances in microbial flora, thereby enhancing the development of the L. japonica industry.”(line418-422)
**Additional Information:**	
**Question**	**Response**
**Financial Disclosure**<br><br>Enter a financial disclosure statement that describes the sources of funding for the work included in this submission. Review the submission guidelines for detailed requirements. View published research articles from *PLOS ONE* for specific examples.<br><br>This statement is required for submission and **will appear in the published article** if the submission is accepted. Please make sure it is accurate.<br><br>**Funded studies**<br>Enter a statement with the following details:<br>• Initials of the authors who received each award<br>• Grant numbers awarded to each author<br>• The full name of each funder<br>• URL of each funder website<br>• Did the sponsors or funders play any role in the study design, data collection and analysis, decision to publish, or preparation of the manuscript?<br><br>Did you receive funding for this work?	Yes
Please add funding details.<br>as follow-up to "**Financial Disclosure**	National key research and development project and sub-project (2021YFD1100504，2021YFD1100504-1，2021YFD1100504-2). Agricultural Science

Powered by Editorial Manager® and ProduXion Manager® from Aries Systems Corporation

Enter a financial disclosure statement that describes the sources of funding for the work included in this submission. Review the submission guidelines for detailed requirements. View published research articles from *PLOS ONE* for specific examples.<br><br>This statement is required for submission and **will appear in the published article** if the submission is accepted. Please make sure it is accurate.<br><br>**Funded studies**<br>Enter a statement with the following details:<br>• Initials of the authors who received each award<br>• Grant numbers awarded to each author<br>• The full name of each funder<br>• URL of each funder website<br>• Did the sponsors or funders play any role in the study design, data collection and analysis, decision to publish, or preparation of the manuscript?<br><br>Did you receive funding for this work?"	and technology independent innovation special key research and development program of Gansu Academy of Agricultural Sciences(2021GARS11). The National Key Research and Development Project and its sub-projects (2021YFD1100504, 2021YFD1100504-1, 2021YFD1100504-2) are national-level scientific research projects on honeysuckle currently being carried out by the project team. These three projects have made significant contributions in terms of personnel allocation, technical support, and financial support.The Agricultural Science and technology independent innovation special key research and development program of Gansu Academy of Agricultural Sciences (2021GARS11) provided Sharing of technical exchanges and experimental bases.
Please select the country of your main research funder (please select carefully as in some cases this is used in fee calculation).<br>as follow-up to "**Financial Disclosure**<br><br>Enter a financial disclosure statement that describes the sources of funding for the work included in this submission. Review the submission guidelines for detailed requirements. View published research articles from *PLOS ONE* for specific examples.<br><br>This statement is required for submission and **will appear in the published article** if the submission is accepted. Please make sure it is accurate.	CHINA - CN

Powered by Editorial Manager® and ProduXion Manager® from Aries Systems Corporation

**Funded studies**<br>Enter a statement with the following details:<br>• Initials of the authors who received each award<br>• Grant numbers awarded to each author<br>• The full name of each funder<br>• URL of each funder website<br>• Did the sponsors or funders play any role in the study design, data collection and analysis, decision to publish, or preparation of the manuscript?<br><br>Did you receive funding for this work?"	
**Competing Interests**<br><br>Use the instructions below to enter a competing interest statement for this submission. On behalf of all authors, disclose any competing interests that could be perceived to bias this work—acknowledging all financial support and any other relevant financial or non-financial competing interests.<br><br>This statement is required for submission and **will appear in the published article** if the submission is accepted. Please make sure it is accurate and that any funding sources listed in your Funding Information later in the submission form are also declared in your Financial Disclosure statement.<br><br>View published research articles from *PLOS ONE* for specific examples.	The authors have declared that no competing interests exist.

Powered by Editorial Manager® and ProduXion Manager® from Aries Systems Corporation

**NO authors have competing interests**<br><br>Enter: *The authors have declared that no competing interests exist.*<br><br>**Authors with competing interests**<br><br>Enter competing interest details beginning with this statement:<br><br>*I have read the journal's policy and the authors of this manuscript have the following competing interests: [insert competing interests here]*<br><br>* typeset	
**Ethics Statement**<br><br>Enter an ethics statement for this submission. This statement is required if the study involved:<br><br>• Human participants<br>• Human specimens or tissue<br>• Vertebrate animals or cephalopods<br>• Vertebrate embryos or tissues<br>• Field research<br><br>Write "N/A" if the submission does not require an ethics statement.<br><br>General guidance is provided below. Consult the submission guidelines for detailed instructions. **Make sure that all information entered here is included in the Methods section of the manuscript.**	no

**Format for specific study types**<br><br>**Human Subject Research (involving human participants and/or tissue)**<br>• Give the name of the institutional review board or ethics committee that approved the study<br>• Include the approval number and/or a statement indicating approval of this research<br>• Indicate the form of consent obtained (written/oral) or the reason that consent was not obtained (e.g. the data were analyzed anonymously)<br><br>**Animal Research (involving vertebrate animals, embryos or tissues)**<br>• Provide the name of the Institutional Animal Care and Use Committee (IACUC) or other relevant ethics board that reviewed the study protocol, and indicate whether they approved this research or granted a formal waiver of ethical approval<br>• Include an approval number if one was obtained<br>• If the study involved *non-human primates*, add *additional details* about animal welfare and steps taken to ameliorate suffering<br>• If anesthesia, euthanasia, or any kind of animal sacrifice is part of the study, include briefly which substances and/or methods were applied<br><br>**Field Research**<br><br>Include the following details if this study involves the collection of plant, animal, or other materials from a natural setting:<br>• Field permit number<br>• Name of the institution or relevant body that granted permission	
**Data Availability**<br><br>Authors are required to make all data underlying the findings described fully available, without restriction, and from the time of publication. PLOS allows rare exceptions to address legal and ethical concerns. See the PLOS Data Policy and FAQ for detailed information.	Yes - all data are fully available without restriction

A Data Availability Statement describing where the data can be found is required at submission. Your answers to this question constitute the Data Availability Statement and **will be published in the article**, if accepted.<br><br>**Important:** Stating 'data available on request from the author' is not sufficient. If your data are only available upon request, select 'No' for the first question and explain your exceptional situation in the text box.<br><br>Do the authors confirm that all data underlying the findings described in their manuscript are fully available without restriction?	
**Describe where the data may be found in full sentences. If you are copying our sample text, replace any instances of XXX with the appropriate details.**<br><br>• If the data are **held or will be held in a public repository**, include URLs, accession numbers or DOIs. If this information will only be available after acceptance, indicate this by ticking the box below. For example: *All XXX files are available from the XXX database (accession number(s) XXX, XXX.).*<br>• If the data are all contained **within the manuscript and/or Supporting Information files**, enter the following: *All relevant data are within the manuscript and its Supporting Information files.*<br>• If neither of these applies but you are able to provide **details of access elsewhere**, with or without limitations, please do so. For example:<br><br>*Data cannot be shared publicly because of [XXX]. Data are available from the XXX Institutional Data Access / Ethics Committee (contact via XXX) for researchers who meet the criteria for access to confidential data.*<br><br>*The data underlying the results presented in the study are available from (include the name of the third party*	All datasets used and/or analyzed during the current study are available within the manuscript.

Powered by Editorial Manager® and ProduXion Manager® from Aries Systems Corporation

and contact information or URL).<br>• This text is appropriate if the data are owned by a third party and authors do not have permission to share the data.<br><br>* typeset	
Additional data availability information:	

Powered by Editorial Manager® and ProduXion Manager® from Aries Systems Corporation

Manuscript

Click here to access/download;Manuscript;Revised Manuscript-12.10.docx

Study on Nitrogen, Phosphorus and Potassium Formula Fertilization Model of *Lonicera japonica Thunb* in hilly and gully Loess Plateau of China

Xiaofeng Jiang *[1,2,3,4], Jianguo Guo *[1], Shangzhong Li [1,2,3,4], Yuan Chen[5], Zhaohui Shi[6], Ping Xu[6], Jiali Chen [5], Qing Fang[5], Yusheng Zhai[6]

[1] Dryland Agriculture Institute / Institute of Plant Protection, Gansu Academy of Agricultural Sciences, Gansu Lanzhou,730070, China;

[2] Key Laboratory of High Water Utilization on Drvland of Gansu Province, Gansu Lanzhou,730070, China;

[3] Key Laboratory of Low-carbon Green Agriculture in Northwestern China,Ministry of Agriculture and Rural Affairs, Lanzhou, Gansu 730070, China;

[4] The Jiont Key Laboratory of Agriculture and Rural Affairs-Gansu Province for Crop Drought Resistance, Yield Increment and Rainwater Efficient Utilization on Rain-fed Area, Lanzhou, Gansu 730070, China;

[5] Agronomy College, Gansu Agricultural University, Gansu Lanzhou, 730070;

[6] Tongwei Qingliang Yuan Limited Liability Company of Lonicerae Japonicae Flos Industry Poverty Alleviation and Development,Gansu Dingxi 743300

[*] Corresponding Author:

Jianguo Guo and Xiaofeng Jiang ; Institute of Plant Protection / Dryland Agriculture Institute , Gansu Academy of Agricultural Sciences, Gansu Lanzhou,730070, China.

Email addresses: jguo1001@163.com; jxf_5188@163.com

Abstract

Formula fertilization is effective in increasing yield and economic profit in medicinal plants, *Lonicera japonica Thunb* (*L. japonica*) is an important medicinal plant, but no study on performance of formula fertilization has been conducted, yet. Thus, the aim of this study is to establish models for higher yield of *L. japonica* by formula fertilization with different ratios of nitrogen (N), phosphorus (P) and potassium (K) in main producing areas in hilly and gully Loess Plateau of China. A '3414' formula fertilization trial with different dose and ratios of N, P, and K used for *L. japonica* was investigated. The fertilizer was poured into a small pit of 30 cm deep around the root of each tree. The spacing between plants and rows was 1.0 m X 1.0 m. The fertilizer model was established by calculating and comparing yield and economic profit of the plant. The results showed application of N, P and K content in soil improved yield effectively. And P was identified as the primary limiting factor for yield. The ratio of output to investment of $N_2P_2K_3$ was lowered significantly. The fertilizer effect function was: $Y = 296.66 - 0.81X_1 + 11.53X_2 + 4.05X_3 - 0.051X_1^2 - 0.60X_2^2 - 0.28X_3^2 + 0.13X_1 \times X_2 + 0.20X_1 \times X_3 - 0.029X_2 \times X_3$. The recommended dose of N, P, and K fertilizer was 18.25-26.75 g, 6.27-8.73 g, and 10.04-13.96 g per plant. By applying formula fertilizer from this study, the yield and economic benefit increased notably by 38.18% and 0.3729 USD per plant. These contribute significantly to production of *L. japonica* with improved yield and profit in specific geographical conditions of cultivating area for economic plant.

Keywords: *L. japonica*; Frequency analysis method; Fertilizer effect function; 3414 experiment; Soil testing formula fertilization.

Introduction

Lonicera japonica Thunb (*L. japonica*), also known as honeysuckle, belongs to the *Caprifoliaceae* family [1]. Gansu Province is the main producing area of

most authentic Chinese medicinal herbs in China. Their buds are harvested and dried before bloom in early summer, which are rich in organic acids, flavonoids, terpenoids, and volatile oils, so they have been widely recognized as effective medicinal herbs for heat-clearing and detoxification, and are commonly used in clinical practice for treating various diseases including pneumonia [2]. Recently, the market demand for honeysuckle is increasing significantly due to the impact of COVID-19. As a result, artificial cultivation has become the primary method of obtaining raw materials [3-4]. However, research on efficient cultivation techniques for honeysuckle is still in its early stages. Therefore, high-quality and efficient production of *L. japonica* is in urgent need to overcome traditional improper fertilization practices.

Formula fertilization is an effective way to improve yield and quality of *L. japonica*[5-6]. Former research found that combined application on N, P and K could not only promote the biosynthesis of Chlorogenic acid and flavonoids in *L. japonica*, but also increase the yield and quality [7-10]. For example, the application of nitrogen fertilizer in Japan enhanced the vegetative growth of *L. japonica*, increased the biological yield of vegetative organs, and elevated the crude protein content of the leaves. However, it resulted in a decrease in both economic yield and chlorogenic acid content of the buds of the reproductive organs [11-14]. Adding P could not only promote flower bud differentiation and increase economic yield, but also increase the Chlorogenic acid content of flower buds and improve commodity quality [15-17]. When the recommended dosages of N, P_2O_5 and K_2O were 34.9 - 47.9, 13.1 - 14.4, 18.5 -23.8 g/plant, the economic yield of *L. japonica* was 387.0 - 430.3 CNY/plant [19].

Tongwei County locates in the hilly and gully region of the Loess Plateau in central Gansu Province. The landform in the territory is composed of hilly and gully like hills, hilly and gully, residual tableland and Sichuan tableland. The mountain ridges are undulating, and the ravines are crisscrossing[20]. The annual

sunshine duration are 1400 h, and the annual total solar radiation is 5446.5 MJ.m^{-2}. There are abundant light and heat resources, which are conducive to the development of modern Silk Road agricultural practices in cold and arid areas. Currently, a high-end *L. japonica* planting base spanning 6,667 ha has been established within the Tongwei Qingliang Yuan Limited Liability Company. This endeavor is part of the Poverty Alleviation and Development initiative in the *L. japonica* Industry. Moreover, the produced *L. japonica* has achieved a remarkable status, both in terms of quality and industry reputation, being at the forefront within China. However, due to long-term planting, there has been a significant increase in fertilizer usage for per tree, which has become a major hindrance to the development of local industries. There is an urgent need to investigate the optimal dosage of N, P, and K fertilizers for *L. japonica*. Therefore, this study conducted an experiment using "3414" Fertilizer Design experiment to establish a model and finally determine a optimal formula for cultivating *L. japonica* with high yield and economic profit.

Materials and Methods

Overview of the test site

The experiment began in April 2022. The experimental site located in Wangjiahe Community, Yaochuan Village, Lidian Township, Tongwei County (1860 m above sea level, 35 ° 25 ′ 40 ″ N, 105 ° 10 ′ 30 ″ E). It belongs to temperate semi-arid Continental climate type, with four distinct seasons, drought and little rain, and abundant light resources. The average annual temperature was 8.2℃, and the annual precipitation was 300 mm, most of them are from July to September. The frost free period was 145 d. The crops mature once a year and belong to the hilly and gully agricultural area of the Loess Plateau in central Gansu, The soil is loam. The irrigation only depended on rainfall, the water, light and other natural factors are consistent. Artificial management of weeding and uniform spraying of pesticides were used during growth period. Imidacloprid

25% wettable powder were sprayed at rate of 24 g a.i. per hectare for the plant.

The experimental site for this experiment was an authorized experimental base of Gansu Agricultural Academy, which has been approved and authorized by the provincial government to serve as an experimental field site. The development of *L. japonica* industry has unique natural advantages. The soil pH of the experimental site was 8.49. The organic matter in soil was 11.01 g/kg. total N was 0.6 g/kg, available N was 42.80 mg/kg. Total P was 0.72 g/kg, available P was 8.97 mg/kg, total K was 17.15 g/kg, and the available K was 160.67 mg/kg.

Materials

The tested *L. japonica* species was Beihua No.1 of 4 year's cultivating time. The fertilizers used in the test were mainly urea (total N content is 46%, Petro China Company Limited), granular Superphosphate (the effective P_2O_5 was 12%, Baoji Fenghe Chemical Co., Ltd.), Potassium sulfate (K_2O was 50%, Qinghai Laishuo Industry and Trade Co., Ltd.).

Method

Design of experiments

This experiment adopted quadratic regression optimal design scheme of '3414' with 3 fertilizer factors set as N, P, and K, and the levels were 0, 1, 2, and 3 [10]. There we[illegible]4 treatments totally. Among them, of which level 0 means with no fertilization; level 2 means the commonly used fertilization amo[illegible] level 1 means 1/2 of the commonly used fertilization amount; and level 3 means 3/2 of the commonly used fertilization amount. The formulized fertilizer was mixed evenly, and the fertilizer were poured into a small pit of 30 cm deep around the root of each tree. This was the first time for formula fertilization experiment carried out on the Tongwei *L. japonica* in the hilly and gully area of the Loess Plateau in central Gansu. There is no reference data for the determination of the 2 levels of each factor (the approximate value of the local optimal fertilization amount). Therefore, it was determined by reference to the local yield level in

2016-2021 and the fertilization amount in Tongwei County. The fertilization dose was 30 g N, 10 g P_2O_5, and 16 g K_2O per plant. Each treatment had 6 *L. japonica* trees, 3 replicates, arranged by random blocks, and the spacing between plants and rows was 1.0 mX1.0 m. Nitrogen fertilizer, phosphorus fertilizer, and potassium fertilizer were applied in a one-time manner on April 8, 2022 before germination. The method of fertilization was ditch application, and the fertilization amount was determined according to Table 1.

Table 1 Experimental design of ‘3414’ test.

Number	Treatment	N-coded value	P-coded value	K-coded value	Fertilization amount (kg.hm^{-2})		
					N	P_2O_5	K_2O
1	$N_0P_0K_0$	0	0	0	0	0	0
2	$N_0P_2K_2$	0	2	2	0	10	16
3	$N_1P_2K_2$	1	2	2	15	10	16
4	$N_2P_0K_2$	2	0	2	30	0	16
5	$N_2P_1K_2$	2	1	2	30	5	16
6	$N_2P_2K_2$	2	2	2	30	10	16
7	$N_2P_3K_2$	2	3	2	30	15	16
8	$N_2P_2K_0$	2	2	0	30	10	0
9	$N_2P_2K_1$	2	2	1	30	10	8
10	$N_2P_2K_3$	2	2	3	30	10	24
11	$N_3P_2K_2$	3	2	2	45	10	16
12	$N_1P_1K_2$	1	1	2	15	5	16
13	$N_1P_2K_1$	1	2	1	15	10	8
14	$N_2P_1K_1$	2	1	1	30	5	8

Measurement items and methods

Yield measurement: the total yield was picked by hand and was calculated in June. The yield measurement was through the manual picking of workers, each person picked six trees, the flower weight of each tree was weighed with a

high-precision electronic balance (model AE001, Jiangsu Hongli weighing Equipment Co., LTD.), and the yield of each tree was recorded. The average value of the flower yield of six trees was the yield of a single tree.

Soil total nitrogen content was determined by NY/T 53-1987 standard Kjeldt nitrogen determination method, soil total phosphorus content was determined by NY/T 88-1988 standard molybdenum-antimony resistance colorimetric method, and soil total potassium content was determined by NY/T 87-1988 standard acid melting flame photometer and FP6450 type flame photometer.

Data analysis

DPS7.5 software and Exce1 2010 software were used to fit the univariate quadratic, binary quadratic and ternary quadratic fertilizer effect functions of yield and fertilizer application, and whether they comply with typical fertilizer efficiency models was analyzed. If it belongs to an atypical fertilizer efficiency model, the frequency analysis method was used to calculate the economic optimal fertilizer amount and the maximum yield fertilizer amount.

The frequency analysis calculation formula was [13, 20]:

$$\overline{x_i} = \frac{1}{n_j}\sum_{j=0}^{p} u_j\, n_{ij}(i = 1,2,3) \qquad (1)$$

In the formula, n_{ij} represents the number of occurrences of the variable value X_{ij}, where (u_j), and n_j represents the total number of occurrences of the variable X_i.

Standard deviation:

$$S\overline{x_i} = \frac{1}{\sqrt{n_j-1}} \cdot \sqrt{\sum_{j=0}^{p}\left(u_j - \overline{x_i}\right)^{\cdot 2} n_{ij}} \qquad (2)$$

Mean standard deviation:

$$S_x = \frac{S}{\sqrt{n_j}} \qquad (3)$$

95% confidence interval：

$$\left(\overline{x_i} - S\overline{x_i} \cdot t_{0.05(n_j-1)}\right) \sim \left(\overline{x_i} + S\overline{x_i} \cdot t_{0.05(n_j-1)}\right) \qquad (4)$$

The economic benefits were calculated as follows:

Output value (CNY/plant) = Yield of per plant*24/1000 (5)

Fertilizer input (CNY/plant) = (nitrogen fertilizer amount of per plant *4.8/1000) + (phosphorus fertilizer amount of per plant *6/1000) + (potass ium fertilizer amount of per plant *8/1000) (6)

Yield to investment ratio (%)= output value /fertilizer input (7)

Result

N and P fertilizers promoting the increase of yield and economic benefits of *L. japonica* per plant

Soil fertility is the basic resource for sustainable agricultural development, nitrogen and phosphorus are the essential nutrients for crop growth and development, nitrogen and phosphorus affect the growth and development of crops, and then affect their yield and economic benefits [18]. Guo Yu et al.'s study on Qianhu found that reasonable distribution of soil nitrogen and phosphorus nutrients can effectively increase the accumulation of soil available nutrients and rhizosphere nutrients, and promote the increase of yield [18]. The index of soil nutrient abundance and deficiency was expressed as the percentage of yield in the nutrient deficient area to the total yield in fertilizer deficient area. That is, the relative yield can express the abundance and deficiency of soil nutrients [5]. Soil nutrients with a relative yield below 50% are extremely low; 50% -75% is low; 75% -95% is medium; greater than 95% is high. Thus, the index of soil nutrient abundance and deficiency of a certain crop in a certain area and the corresponding fertilizer amount were determined [19-20]. It is high to determine the soil nutrient abundance and deficiency index of a certain crop in a certain area and the corresponding fertilization amount. From Table 2, it can be seen that the contribution rate of basic soil fertility in the field trial site is 73.97%. The yield in the non fertilized area accounts for 73.97% of the total yield in the fully fertilized area, the yield in the N deficient area accounts for 84.67% of the total

yield in the fully fertilized area, the yield in the P deficient area accounts for 78.70% of the total yield in the fully fertilized area, and the yield in the K deficient area accounts for 80.72% of the total yield in the fully fertilized area. The findings indicated that the soil's available nitrogen, phosphorus, and potassium content fell within the moderate range, with the soil's capacity to supply these nutrients being at an intermediate level. Therefore, the P content of soil is the primary fertility factor that limits the yield of *L. japonica*. Within the recommended fertilization range, each application with 1 g of N, P, and K fertilizer can increase the yield of buds by 2.04 g, 8.52 g, and 4.82 g, respectively. The results showed that increasing P fertilizer had a significant effect on improving yield of *L. japonica* buds.

Table 2 The relative yield and increasing rate of fertilizer deficiency treatments

Processing number		Yield (g/plant)	Relative yield (%)	Increased yield of 1g fertilizer production of bud fresh weight（g）		
				N	P_2O_5	K_2O
1	$N_0P_0K_0$	295.77	73.97	-	-	-
2	$N_0P_2K_2$	338.53	84.67	2.04	-	-
4	$N_2P_0K_2$	314.67	78.70	-	8.52	-
8	$N_2P_2K_0$	322.73	80.72	-	-	4.82
6	$N_2P_2K_2$	399.83	100.00	-	-	-

From Table 3, it can be seen that the yield of *L. japonica* per plant was greatly affected by different treatments. Among them, the yield and economic benefits of $N_2P_3K_2$ treatment were the highest at 409 g per plant and 1.3499 USD per plant. The treatment of $N_2P_3K_2$ increased the yield significantly by 38.18% compared to the control, while the yield and economic benefits of the non fertilized control were the lowest on 296 g per plant and 0.9770 USD per plant. The highest output to investment ratio was the treatment of $N_1P_2K_1$, which reached 47.26. However, the lowest output ratio for output to investment ratio was $N_2P_2K_3$, which was 23.96. For treatments of $N_0P_2K_2$, $N_1P_2K_2$, $N_2P_2K_2$,

$N_3P_2K_2$, and $N_2P_0K_2$, $N_2P_1K_2$, $N_2P_2K_2$, and $N_2P_3K_2$ in Table 3,With the increase of nitrogen and phosphate fertilizer amount, the yield per plant of *L. japonica* increased gradually. From the treatments of $N_2P_2K_2$, $N_2P_2K_0$, $N_2P_2K_1$, and $N_2P_2K_3$ in From Table 2, it can be seen that as rate of P increases in production, the yield increased at low ratios and reached its highest value at treatment of No.6. However, when the amount of P application continued to increase until to treatment 10, the yield of per plant did not increase any more, but decreased slightly. The results of interaction relationship of fertilizer was PK>NP>NK, and the yield increased 14.46%, 9.12% and 6.39%, respectively compared with treatment with no fertilizer.

Table 3 Effects of different treatments on *L. japonica* yield and economic benefits

Experiment number		Yield（g/plant）	Increased profit than control (%)	Economic profit (CNY/ plant)	Fertilizer cost (CNY/ plant)	Ratio of profit to investment
1	$N_0P_0K_0$	295.77 ± 2.85j	-	7.10	0.000	-
2	$N_0P_2K_2$	338.5 ± 2.21h	14.46	8.12	0.188	43.22
3	$N_1P_2K_2$	389.7 ± 5.30c	31.76	9.35	0.260	35.97
4	$N_2P_0K_2$	314.67 ± 9.08i	6.39	7.55	0.272	27.76
5	$N_2P_1K_2$	378.5 ± 10.41de	27.97	9.08	0.302	30.08
6	$N_2P_2K_2$	399.83 ± 8.13ab	35.19	9.60	0.332	28.90
7	$N_2P_3K_2$	408.70 ± 2.86a	38.18	9.81	0.362	27.10
8	$N_2P_2K_0$	322.73 ± 1.16i	9.12	7.75	0.204	37.97
9	$N_2P_2K_1$	372.27 ± 4.92ef	25.86	8.93	0.268	33.34
10	$N_2P_2K_3$	395.27 ± 1.68bc	33.64	9.49	0.396	23.96
11	$N_3P_2K_2$	407.93 ± 4.14a	37.92	9.79	0.404	24.23
12	$N_1P_1K_2$	367.80 ± 4.78f	24.35	8.83	0.230	38.38
13	$N_1P_2K_1$	386.73 ± 5.14cd	30.76	9.28	0.196	47.36
14	$N_2P_1K_1$	353.4 ± 10.83g	19.49	8.48	0.238	35.64

Note: The ‘-‘ indicates does not exist, the unit price of *L. japonica* is 3.3024 USD/kg, N is 0.6605 USD/ kg,

P_2O_5 is 0.8256 USD/kg, and $K_2O$is 1.1008 USD/kg·

Analysis of fertilizer effect function

Analysis of Ternary Quadratic Fertilizer Effect Function

Using DPS software to perform regression analysis between independent variables x and y (yield per plant), the fertilizer effect function was calculated as: $Y=296.66-0.81X_1+11.53X_2+4.05X_3-0.051X_1^2-0.60X_2^2-0.28X_3^2+0.13X_1*X_2+0.20X_1*X_3-0.029X_2*X_3$; where 0.81, 11.53 and 4.05 are the absolute values of the first term of the equation, respectively. The results showed that the yield of *L. japonica* was affected by the single factor of N, P, and K in the order of P > K > N. Indicating that it can be seen that P fertilizer had the greatest impact on the yield of honeysuckle, while N fertilizer had the smallest effect. This result was consistent with results in Table 2.

The correlation coefficient R^2 was 0.9938 ($P=0.0018<0.05$) obtained by using DPS software for multiple regression analysis and test. It passed F-test and reached a significant level. It showed that there was a high correlation between independent variables x and y (yield). and there was a significant regression relationship between yield of *L. japonica* and N, P, K fertilizers, but the coefficient of the first term in the equation had both positive and negative values, which did not conform to the typical ternary quadratic fertilizer effect function. The calculation of recommended fertilization amount by using the marginal derivative method was not consistent with actual production. Therefore, frequency analysis method is used to calculate the recommended fertilization amount.

Analysis of single factor fertilizer effect function Single factor refers to fixing the fertilizer level of two factors and the relationship equation between fertilizer change and yield change was fitted. From Table 2, it can be seen that the fitting of the single factor fertilizer effect function was the same as that of the 3 factors, and the fertilizer usage and yield of N, P, and K fertilizers were applied for fitting. Regression analysis was conducted on N fertilizer effect function and correlation

coefficients with F and P values using treatments N numbered 2, 3, 6, and 11 in Table 1. Similarly, the effect equations of P fertilizer were fitted using treatments 4, 5, 6, and 7, while the effect equations of K fertilizer were fitted using treatments 6, 8, 9, and 10. The effect equations of each fertilizer are detailed in Table 2. The results showed that the correlation coefficient R of the fertilizer effect function of N fertilizer was 0.9868 (P=0.1621>0.05), and the regression effect of the equation was not significant. The correlation coefficient R of the fertilizer effect function of P fertilizer was 0.9955, (P=0.0944>0.05), and the regression effect of the equation is not significant. The correlation coefficient R of the fertilizer effect function of K fertilizer is 0.9990 (P=0.0440<0.05), and the regression effect of the equation is significant.

From Table 4 and Table 5, it can be seen that the maximum fertilization amounts of N, P_2O_5, and K_2O for each factor in the single factor fertilizer effect function are 7.5, 13, and 19.29, respectively. The corresponding maximum fertilization amounts are 408.45 g/ plant, 409.03 g/plant, and 445.99 g/plant, respectively.

Table 4 Analysis on single factor fertilizer effect function

Factor	Fertilizer effect function	R^2	P
N fertilizer	$Y=340.95+3.60X_1-0.048X_1^2$	0.9868	0.1621
P fertilizer	$Y=316.55+14.31X_2-0.55X_2^2$	0.9955	0.0944
K fertilizer	$Y=322.40+8.11X_3-0.21X_3^2$	0.9990	0.0440

Table 5 Single factor fertilizer effect function each factor fertilizer application and maximum yield analysis

Factor functions	Maximum fertilization amount of each factor（g/plant）	Maximum output（g/plant）
Fertilizer effect function of N	7.5	408.45
Fertilizer effect function of P	13	409.03
Fertilizer effect function of K	19.29	445.99

Analysis of Two-Factor Fertilizer Effect Function

The two-factor fertilizer equation was an equation that fixes level of one

fertilizer unchanged and fits the changes in the levels of two fertilizers with the changes in yield. The fitting of the interaction effect function between the two factors was the same as that of the three factors. From Table 6, it can be seen that the regression analysis was performed on the fertilizer dosage and yield through NP, NK, and PK fertilizer related treatments. The interaction effect equation of NP fertilizer was obtained through regression analysis between the fertilizer dosage and yield of treatments numbered 2, 3, 4, 5, 6, 7, 11, and 12 in Table 1. Similarly, the interaction effect equation of NK fertilizer was obtained through regression analysis of fertilizer dosage 2 and yield under treatments of 2, 3, 6, 8, 9, 10, 11, and 13. The interaction effect equation of PK fertilizer was obtained through regression analysis of fertilizer dosage 2 and yield under treatments of 4, 5, 6, 7, 8, 9, 10, and 14. The results showed that the correlation coefficient R^2 of the fertilizer effect function of NP fertilizer was 0.9946, $P=0.0269<0.05$, indicating a significant regression effect of the equation; The correlation coefficient R^2 of the fertilizer effect function of NK fertilizer was 0.9898, $P=0.0498<0.05$, and the regression effect of the equation was significant; The correlation coefficient R^2 of the fertilizer effect function of PK fertilizer was 0.9948, $P=0.0259<0.05$, and the equation regression effect was significant.

Table 6 Analysis on fertilizer effect function of two factors

Factor	Fertilizer effect function	R^2	P
NP fertilizer	$Y=303.10+1.94X_1+9.72X_2-0.051X_1^2-0.60X_2^2+0.18X_1*X_2$	0.9946	0.0269
NK fertilizer	$Y=382.48-0.80X_1+1.35X_3-0.042X_1^2-0.24X_3^2+0.25X_1*X_3$	0.9898	0.0498
PK fertilizer	$Y=260.41+11.11X_2+7.36X_3-0.50X_2^2-0.24X_3^2+0.15X_2*X_3$	0.9948	0.0259

Production frequency analysis

According to the above analysis, the regression effects of N and P fertilizers were not significant in the single factor fertilizer effect function, while the regression effects of K fertilizers were significant. The regression effects of NP, NK, and PK fertilizers were significant in the fertilizer effect functions of the interaction

of two factors. The ternary quadratic fertilizer effect function was not a typical ternary quadratic fertilizer effect function. Therefore, this study was not suitable for using the marginal derivative method to calculate the maximum yield fertilization amount and the optimal fertilization amount. For results that did not fit the typical ternary quadratic fertilizer effect function, the yield frequency analysis method could be used to determine the appropriate recommended fertilization amount.

Setting Statistical Intervals (Yield Range)

According to the results of the field experiment (Table 7), the yield range was set. The maximum actual average yield of this experimental community was 409 g/plant, and the minimum was 296 g/plant. The yield range (statistical interval) was set to $296<y<409$. Frequency analysis was calculated based on the field yield statistical table of *L. japonica*.

Table 7 Field trial yield of *L. japonica*

Number	Treatment	Nutrient consumption (g.plant $^{-1}$)			Yield per plant (g.plant $^{-1}$)			
		N	P_2O_5	K_2O	I	II	III	Average value
1	$N_0P_0K_0$	0	0	0	297	296	295	296
2	$N_0P_2K_2$	0	10	16	337	338	340	339
3	$N_1P_2K_2$	15	10	16	386	396	387	390
4	$N_2P_0K_2$	30	0	16	313	307	325	315
5	$N_2P_1K_2$	30	5	16	376	370	390	379
6	$N_2P_2K_2$	30	10	16	391	402	407	400
7	$N_2P_3K_2$	30	15	16	406	412	408	409
8	$N_2P_2K_0$	30	10	0	324	323	322	323
9	$N_2P_2K_1$	30	10	8	370	369	378	372
10	$N_2P_2K_3$	30	10	24	395	397	394	395
11	$N_3P_2K_2$	45	10	16	407	413	405	408

12	$N_1P_1K_2$	15	5	16	364	373	366	368
13	$N_1P_2K_1$	15	10	8	390	381	390	387
14	$N_2P_1K_1$	30	5	8	359	341	360	353

Yield frequency analysis

The frequency of each factor level of nitrogen, phosphorus and potassium falling into the set yield range was counted respectively, and the average, standard deviation, and 95% confidence interval recommended fertilization amount of nitrogen, phosphorus, and potassium according to formulas (1) - (4) were calculated to obtain the yield frequency analysis of honeysuckle, as shown in Table 8. By calculation, the times of nitrogen fertilizer yield falling into the set interval at 0-3 level were 5, 9, 21 and 2 times, the times of phosphate fertilizer yield falling into the set interval at 0-3 level were 5,9,24 and 3 times, and the times of potassium fertilizer yield falling into the set interval at 0-3 level were 5,9,24 and 3 times, respectively. The average fertilization rates of nitrogen, phosphorus, and potassium fertilizers were 23.57,7.86 and 12.57, respectively. The recommended fertilization amount for nitrogen fertilizer (N) is 18.25-26.75 g/plant, for phosphorus fertilizer (P_2O_5) is 6.27-8.73 g/plant, and for potassium fertilizer (K_2O) is 10.04-13.96 g/plant, resulting in a yield of 376.45-397.94 g/plant.

Table 8 Frequency analysis of *L. japonica* output

	N fertilization amount			P fertilization amount			K fertilization amount		
Fertilization level	Fertilization amount (g/plant)	Frequency	Rate(%)	Fertilization amount (g/plant)	Frequency	Rate (%)	Fertilization amount (g/plant)	Frequency	Rate (%)
0	0	5	13.51	0	5	12.20	0	5	12.20
1	15	9	24.32	5	9	21.95	8	9	21.95
2	30	21	56.76	10	24	58.54	16	24	58.54
3	45	2	5.41	15	3	7.32	24	3	7.32
Sum of times		37			41			41	
x_i average value	22.5			7.5			12		
Standard deviation	13.17			4.05			6.40		

(s)			
Standard deviation	2.17	0.63	1.00
95%confidence interval	18.25-26.75	6.27-8.73	10.04-13.96

Calculation and Optimization of Fertilization Rate and Yield

The average application rates of nitrogen, phosphorus, and potassium fertilizers obtained by frequency analysis was 22.5 g/plant, 7.5 g/plant, and 12 g/plant, which were brought into the ternary quadratic fertilizer effect function, and the optimized fertilization yield was calculated to be 386.95 g/plant.

Discussion

Nitrogen, phosphorus, and potassium are the three primary nutrient elements critical for plant growth and development, exhibiting interactive effects within the plant body. Nitrogen plays a key role in the synthesis of proteins, nucleic acids, and chlorophyll, thereby promoting photosynthesis and enhancing both the yield and quality of crops. Phosphorus, an essential component of nucleic acids and nuclear proteins, increases the number of buds and the thousand-grain weight, ultimately contributing to improved yields in honeysuckle. Potassium is absorbed by plant roots in the form of ions and plays a crucial role in fundamental metabolic processes, thereby influencing plant growth and development as well as enhancing crop stress resistance. When nitrogen, phosphorus, and potassium fertilizers are applied to the soil, the various nutrient elements interact with soil microorganisms and organisms, promoting mutual enhancement of their effects. This interaction maximizes the efficacy of nitrogen, phosphorus, and potassium fertilizers, ultimately improving fertilizer utilization rates and enhancing both crop yield and quality [22-23].In this study, the yield of L. japonica increased with the increase of nitrogen and phosphorus content.

Inorganic elements such as nitrogen (N), phosphorus (P), and potassium (K) are the mineral elements with the highest demand for plant metabolism, tissue

and organ formation, and overall growth and development. Fertilization has been employed in agricultural production to satisfy the requirements of plants for these three essential elements [24]. Cao et al. [25] demonstrated that balanced fertilization of N, P, and K significantly enhances plant growth and development, as evidenced by increases in the number of flower nodes, fresh weight of flowers, and the number of flower buds, ultimately improving flower bud yield.Research has demonstrated that fertilization can enhance the yield of *L. japonica*. As the level of fertilization increases, the yield per plant also rises. However, when the amount of fertilization surpasses a certain threshold, the yield experiences a significant decline [26, 27].A single experiment conducted on nitrogen (N), phosphorus (P), and potassium (K) revealed that the order of their effect on increasing the yield of *L. japonica* is as follows: N > P > K. The optimal application rates for these nutrients were determined to be 0.1 kg/plant for N, 0.2 kg/plant for P, and 0.1 kg/plant for K, respectively [28].The results of the yield study indicated that nitrogen (N), phosphorus (P), potassium (K), and compound fertilizers can enhance the differentiation of flower buds in honeysuckle plants, which is positively correlated with the length of the flower buds.Among these elements, nitrogen (N) has the potential to enhance the yield of both leaves and flowers. In contrast, phosphorus (P) serves as the primary fertility factor influencing flower bud weight and yield, playing a crucial role in the growth and development of flower buds [29].In this study, varying fertilizer ratios significantly influenced the yield of *L. japonica* per plant. Specifically, the yield for treatment $N_2P_3K_2$ increased by 38.18% compared to the control. Additionally, the contribution rate of basic soil fertility at the field trial site was found to be 73.97%.Within the recommended range for fertilization, the application of 1 g of nitrogen, phosphorus, and potassium fertilizer has the potential to increase the yield of *L. japonica* buds by 2.04 g, 8.52 g, and 4.82 g, respectively.The results indicated that phosphorus (P) fertilizer was the primary limiting factor for

enhancing the yield of *L. japonica* buds. Therefore ,it is essential to consider the amount of phosphorus fertilizer applied to achieve high yields and a finding that aligns with the results of Zeng Huijie et al. [24]. Tongwei County locates in the hilly and gully region of the Loess Plateau in central Gansu, the correlation coefficient (R^2) of the equation function representing the fertilizer effect of *L. japonica* was established through regression analysis. This analysis was based on the yield of *L. japonica* obtained from experiments and the amounts of nitrogen (N), phosphorus (P), and potassium (K). The resulting R^2 value was 0.9938, with a p-value of 0.0018, which is less than 0.05, indicating a significant regression effect.However, the coefficients of the first terms were -0.81, 11.53, and 4.05, respectively. These values represent typical fertilizer effect functions and do not conform to the law of diminishing returns.The F-test yielded a p-value of 0.0018, which is less than the significance level of 0.05, indicating a statistically significant result. Additionally, the marginal analysis method was employed to determine the optimal fertilization amount and the maximum yield fertilization amount; however, these findings were inconsistent with actual production levels.The recommended fertilization amount for 4-year-old *L. japonica*, determined through frequency analysis, was as follows: nitrogen fertilizer (N) should be applied at a rate of 18.25-26.75 g per plant. The recommended amount for phosphorus fertilizer (P_2O_5) is 6.27-8.73 g per plant, while for potassium fertilizer (K_2O), the suggested amount ranges from 10.04 to 13.96 g per plant. The expected yield per plant is between 376.45 and 397.94 g.The conventional fertilization amounts, based on local yield levels and data from Tongwei County between 2016 and 2021, were determined to be 30 g/plant of pure nitrogen, 10 g/plant of P_2O_5, and 16 g/plant of K_2O. These amounts were found to be higher than the recommended fertilization levels derived from frequency analysis. Consequently, the recommended fertilization amount for the upcoming year's tests should be adjusted to a slightly lower level.

In summary, due to soil texture, and fertility, fertilization strategies should be tailored to the specific conditions of each planting region. Additionally, it is essential to ensure a balanced application of inorganic fertilizers to prevent soil compaction and imbalances in microbial flora, thereby enhancing the development of the *L. japonica* industry.

Conclusion

This study demonstrated that the application of formula fertilization with varying ratios of nitrogen (N), phosphorus (P), and potassium (K) can significantly enhance the yield of fresh buds of *L. japonica* in areas of China characterized by poor soil nutrient availability. The use of formula fertilizer containing N, P, and K improved soil nutrient levels to a medium grade, with phosphorus content identified as the primary limiting factor affecting yield.Increasing the doses of nitrogen (N), phosphorus (P), and potassium (K) fertilizers to the recommended levels resulted in a significant enhancement in yield. Consequently, the yield per plant reached 409 g, yielding an economic benefit of 1.3499 USD per gram per plant. This represents a substantial increase of 38.18% in yield, along with a notable economic benefit of 0.3729 USD per gram per plant compared to the control group. To model this effect, the following function was derived: $Y=296.66-0.81X_1+11.53X_2+4.05X_3-0.051X_1^2-0.60X_2^2-0.28X_3^2+0.13X_1\times X_2+0.20X_1 \times X_3-0.029X_2 \times X_3$. This study examines the combined application of N, P_2O_5, and K_2O fertilizers per plant. Through frequency analysis, the recommended doses of N, P_2O_5, and K_2O fertilizers were determined to be within the ranges of 18.25 to 26.75 g/plant, 6.27 to 8.73 g/plant, and 10.04 to 13.96 g/plant, respectively. The findings of this research have practical implications for the cultivation and production of *L. japonica*, offering valuable insights for optimizing cultivation practices and maximizing the potential of this important medicinal plant within the specific geographical

conditions of the cultivation area.

Funding

National key research and development project and sub-project (2021YFD1100504，2021YFD1100504-1，2021YFD1100504-2). Agricultural Science and technology independent innovation special key research and development program of Gansu Academy of Agricultural Sciences(2021GARS11).

the Gansu Provincial Agricultural Science and Technology Special Project（GNKJ-2021-33）, the National Natural Science Foundation of China (32260550).The National Key Research and Development Project and its sub-projects (2021YFD1100504, 2021YFD1100504-1, 2021YFD1100504-2) are national-level scientific research projects on honeysuckle currently being carried out by the project team, These three projects have made significant contributions in terms of personnel allocation, technical support, and financial support.The Agricultural Science and technology independent innovation special key research and development program of Gansu Academy of Agricultural Sciences (2021GARS11),the Gansu Provincial Agricultural Science and Technology Special Project（GNKJ-2021-33）and the National Natural Science Foundation of China (32260550) provided Sharing of technical exchanges and experimental bases.

Conflicts of Interest

The authors have no conflicts of interest to declare.

Availability of data and materials

All datasets used and/or analyzed during the current study are available within the manuscript.

References：

1. Liu Z, Cheng Y, Chao Z. A Comprehensive Quality Analysis of Different Colors of Medicinal and Edible Honeysuckle. Foods. 2023;12(16):

3126. https://doi.org/10.3390/foods12163126

2. Xiao O, Li M, Chen D, Chen J, Simal-Gandara J, Dai X, et al. The dissipation, processing factors, metabolites, and risk assessment of pesticides in honeysuckle from field to table. Journal of Hazardous Materials. 2022;431:128519. https://pubmed.ncbi.nlm.nih.gov/35231811/

3. Qin S, Chen X, Jiang C, Li M, Yuan Y, Yang J, et al. Pruning induced yield and quality variations and the correlated gene expression and phytohormone changes in Lonicera japonica. Industrial Crops and Products. 2019;132:386-95. https://www.cabidigitallibrary.org/doi/full/10.5555/20193253630

4. Ivona E, Mirela C, Emil Ch,Madalina B,Monica S,Mihaela S.Influence of cultivar and fertilization with vermicompost on fruit quality and yield in lonicera spp. Current Trends in Natural Sciences,2021,10(19):72-79. https://doi.org/10.47068/ctns.2021.v10i19.009

5. Guo R,Liu R Y, Huang H Y, Zhai Y Sh,Chen Y,liW D. Effects of Different Fertilization Formulas on Yield and Quality of LoniceYa japonica Flos and Soil Microecology in Loess Plateau. Journal of Nuclear Agricultural Sciences.2023,37(7):1452-1461. https://doi.org/10.11869/j.issn.10008551.2023.07.1452

6. Peng X P,Zhang J Y,Su Y Y,Sun L Q,Liu X H,Tang X Q,et al. Effect s of spraying copper and borax fertilizer on growth physiology of Lo nicera japonica Thunb.and quality of Lonicera japonica flos .Journal o f Nanjing Agricultural University,2023,46(1):33-41. https://doi.org/10 . 7 685/jnau . 202202004

7. Zhang X, Li S, An X, Song Z, Zhu Y, Tan Y, et al. Effects of nitro gen, phosphorus and potassium formula fertilization on the yield and berry quality of blueberry. Plos one. 2023;18(3):e0283137. https://doi.o rg/10.1371/journal.pone.0283137

8. Zhao F, Li F, Zhou J, Sun X, Wang Y, Jing L, et al. Soiltesting formula fertilization with organic fertilizer addition for target yield cannot stand long due to stem lodging of rice. Frontiers in Plant Science.2022;13:1091156. https://doi.org/10.3389/fpls.2022.1091156
9. Bao Q-p, Han X-r, Cui Z-g, Yin Y, Zhang H, Li W-r, et al. Recom mended fertilization model for spring maize nitrogen fertilizer in Nort heast China. Journal of Plant Nutrition and Fertilizers. 2020;26(4):705-16. https://10.11674/zwyf.19247
10. Saquee FS, Diakite S, Kavhiza NJ, Pakina E, Zargar M. The Efficacy of Micronutrient Fertilizers on the Yield Formulation and Quality of Wheat Grains. Agronomy. 2023;13(2):566. https://doi.org/10.3390/agronomy13020566
11. Guo L, Qiao J, Zhou D, Qin D, Huo J. Effect of Vermicompost on Fruit Quality, Growth, and Rhizosphere Soil Enzyme Activities of Blue Honeysuckle (Lonicera caerulea L.). Journal of Soil Science and Plant Nutrition. 2023:1-9. https://springer.longhoe.net/article/10.1007/s42729-023-01300-9
12. Guo R, Liu R Y, Huang H Y, Zhai Y Sh, Chen Y, Li W D. Effects of Different Fertilization Formulas on Yield and Quality of Lonicer a japonica Flos and Soil Microecology in Loess Plateau. Journal of N uclear Agricultural Sciences,2023,37(7):1452~1461. https://doi10.11869/j. issn.1000-8551.2023.07.1452.
13. Chen Y, Zhou X, Ma L, Lin Y, Huang X. Chinese yam yield is affected by soil nutrient levels and interactions among N, P, and K fertilizers. Chinese Herbal Medicines. 2023. https:// pii/S1674638423000448
14. Liao G, Wang Y, Yu H, He P, Lin Z, Dai T, et al. Nutrient use effic iency has decreased in southwest China since 2009 with increasing ris k of nutrient excess. Communications Earth & Environment. 2023;4(1):

388. https://www.nature.com/articles/s43247-023-01036-5

15. Iseki K, Ikazaki K, Batieno BJ. Heterogeneity effects of plant density and fertilizer application on cowpea grain yield in soil types with different physicochemical characteristics. Field Crops Research. 2023;292:108825. https://doi.org/10.1016/j.fcr.2023.108825

16. Yang L, Lin Y, Kong J, Yu Y, He Q, Su Y, et al. Effects of fertilization and dry-season irrigation on the timber production and carbon storage in subtropical Eucalyptus plantations. Industrial Crops and Products. 2023;192:116143. https://doi.org/10.1016/j.indcrop.2022.116143

17. Tang S, Zhou J, Pan W, Sun T, Liu M, Tang R, et al. Effects of combined application of nitrogen, phosphorus, and potassium fertilizers on tea (Camellia sinensis) growth and fungal community. Applied Soil Ecology. 2023;181:104661. https://doi.org/10.1016/j.apsoil.2022.104661

18. Guo Y, Xu G, Luo Sh, Luo M,Tan Q Sh,Yang Y D, et al.Effects of nitr ogen,phosphorus and potassium mixed fertilizer application on yield and quality dynamic of *peuce-danum praeruptorum* Dunn. Chinese soil andf ertilizer,2023,(10):169-176.https://doi.org/10.11838/sfsc.1673-6257.22539.

19. Fontes PCR, Braun H, Busato C, Cecon PR. Economic optimum nitro gen fertilization rates and nitrogen fertilization rate effects on tuber ch aracteristics of potato cultivars. Potato Research. 2010;53:167-79. https: //springer.longhoe.net/article/10.1007/s11540-010-9160-3

20. Li H, Zhang Y, Sun Y, Liu P, Zhang Q, Wang X, et al. Long-term effects of optimized fertilization, tillage and crop rotation on soil fertility, crop yield and economic profit on the Loess Plateau. European Journal of Agronomy. 2023;143:126731. https://doi.org/10.1016/j.eja.2022.126731

21. Niu Sh W,Wang Zh F, Li G Zh, Ma L B. Analyzing the Potentials and Structure of Rural Household Energy Use in Hilly Regions of the Loess Plateau：A Case Study in Qizuei Village of Tongwei County o

f Gansu Province. Resources Science. 2007,29(3):105-110. https://doi.org/1007-7588(2007)03-0105-06

22. Wang Q, Wang X-c, Li X-y, Wang B, Gao X-q, Fu B-z. Effect of “3414” Fertilization on Seed Yield and Quality of Bromus inermis in Arid Area of Ningxia. Acta Agrestia Sinica. 2022,30(12):3470. http://doi.org/10.48130/grares-0024-0007
23. XIONG Y, LIU H CH, WANG YP, PAN F G. Effects of Nitrogen, Phosphorus, and Potassium Dosage and Ratio on Seed Yield and Quality of "Qianhuo No. 1”[J] .Journal of Shandong Agricultural University(Natural Science Edition), 2024,55(1):009-015. https://www.cnki.net/KCMS
24. Zeng H, Wang X, Qiao Z, Li Y, Cai N, Liu S, et al. Influence on yield and quality of Lonicera japonica by soil testing and formulated fertilization. J Nucl Agric Sci. 2017;31:2443-9.
25. Cao Y, Pan Y, Wang M, Liu T, Meng X, Guo S. The effects of diff erent nitrogen forms on chlorophyll fluorescence and photosystem II i n Lonicera japonica. Journal of Plant Growth Regulation. 2023;42(7):4 106-17. https://springer.longhoe.net/article/10.1007/s00344-022-10873-1
26. Gardner Z. Effects of Fertilization and Drying Conditions on the Quality of Selected Chinese Medicinal Plants. 2019. https://orcid.org/0000-0001-5351-5276
27. Chen K, Ma L, Chen C, Liu N, Wang B, Bao Y, et al. Long-Term Impact of N, P, K Fertilizers in Different Rates on Yield and Quality of Anisodus tanguticus (Maxinowicz) Pascher. Plants. 2023;12(11):2102.
28. Jourdan C, Silva EV, Gonçalves JLM, Ranger J, Moreira RM, Laclau JP. Fine root production and turnover in Brazilian Eucalyptus plantations under contrasting nitrogen fertilization regimes. Forest ecology and management. 2008;256(3):396-404. https://link.springer.com/article/10.1007/s11368-013-0718-y

29. Zeng P, Guo Z, Xiao X, Peng C, Liu L, Yan D, et al. Physiological stress responses, mineral element uptake and phytoremediation potential of Morus alba L. in cadmium-contaminated soil. Ecotoxicology and environmental safety. 2020;189:109973. https://doi: 10.1016/j.ecoenv.2019.109973

Revised Manuscript with Track Changes

Study on Nitrogen, Phosphorus and Potassium Formula Fertilization Model of *Lonicera japonica Thunb* in hilly and gully Loess Plateau of China

Xiaofeng Jiang *[1,2,3,4], Jianguo Guo *[1], Shangzhong Li [1,2,3,4], Yuan Chen[5], Zhaohui Shi[6], Ping Xu[6], Jiali Chen [5], Qing Fang[5], Yusheng Zhai[6]

[1] Dryland Agriculture Institute / Institute of Plant Protection, Gansu Academy of Agricultural Sciences, Gansu Lanzhou,730070, China;

[2] Key Laboratory of High Water Utilization on Drvland of Gansu Province, Gansu Lanzhou,730070, China;

[3] Key Laboratory of Low-carbon Green Agriculture in Northwestern China,Ministry of Agriculture and Rural Affairs, Lanzhou, Gansu 730070, China;

[4] The Jiont Key Laboratory of Agriculture and Rural Affairs-Gansu Province for Crop Drought Resistance, Yield Increment and Rainwater Efficient Utilization on Rain-fed Area, Lanzhou, Gansu 730070, China;

[5] Agronomy College, Gansu Agricultural University, Gansu Lanzhou, 730070;

[6] Tongwei Qingliang Yuan Limited Liability Company of Lonicerae Japonicae Flos Industry Poverty Alleviation and Development,Gansu Dingxi 743300

[*] Corresponding Author:

Jianguo Guo and Xiaofeng Jiang ; Institute of Plant Protection / Dryland Agriculture Institute , Gansu Academy of Agricultural Sciences, Gansu Lanzhou,730070, China.

Email addresses: jguo1001@163.com; jxf_5188@163.com

Abstract

Formula fertilization is effective in increasing yield and economic profit in medicinal plants, *Lonicera japonica Thunb* (*L. japonica*) is an important medicinal plant, but no study on performance of formula fertilization has been conducted, yet. Thus, the aim of this study is to establish models for higher yield of *L. japonica* by formula fertilization with different ratios of nitrogen (N), phosphorus (P) and potassium (K) in main producing areas in hilly and gully Loess Plateau of China. A '3414' formula fertilization trial with different dose and ratios of N, P, and K used for *L. japonica* was investigated. The fertilizer was poured into a small pit of 30 cm deep around the root of each tree. The spacing between plants and rows was 1.0 m X 1.0 m. The fertilizer model was established by calculating and comparing yield and economic profit of the plant. The results showed application of N, P and K content in soil improved yield effectively. And P was identified as the primary limiting factor for yield. The ratio of output to investment of $N_2P_2K_3$ was lowered significantly. The fertilizer effect function was: $Y = 296.66 - 0.81X_1 + 11.53X_2 + 4.05X_3 - 0.051X_1^2 - 0.60X_2^2 - 0.28X_3^2 + 0.13X_1{\times}X_2 + 0.20X_1{\times}X_3 - 0.029X_2{\times}X_3$. The recommended dose of N, P, and K fertilizer was 18.25-26.75 g, 6.27-8.73 g, and 10.04-13.96 g per plant. By applying formula fertilizer from this study, the yield and economic benefit increased notably by 38.18% and 0.3729 USD per plant. These contribute significantly to production of *L. japonica* with improved yield and profit in specific geographical conditions of cultivating area for economic plant.

Keywords: *L. japonica*; Frequency analysis method; Fertilizer effect function; 3414 experiment; Soil testing formula fertilization.

Introduction

Lonicera japonica Thunb (*L. japonica*), also known as honeysuckle, belongs to the *Caprifoliaceae* family [1]. Gansu Province is the main producing area of

most authentic Chinese medicinal herbs in China. Their buds are harvested and dried before bloom in early summer, which are rich in organic acids, flavonoids, terpenoids, and volatile oils, so they have been widely recognized as effective medicinal herbs for heat-clearing and detoxification, and are commonly used in clinical practice for treating various diseases including pneumonia [2]. Recently, the market demand for honeysuckle is increasing significantly due to the impact of COVID-19. As a result, artificial cultivation has become the primary method of obtaining raw materials [3-4]. However, research on efficient cultivation techniques for honeysuckle is still in its early stages. Therefore, high-quality and efficient production of *L. japonica* is in urgent need to overcome traditional improper fertilization practices.

Formula fertilization is an effective way to improve yield and quality of *L. japonica*[5-6]. Former research found that combined application on N, P and K could not only promote the biosynthesis of Chlorogenic acid and flavonoids in *L. japonica*, but also increase the yield and quality [7-10]. For example, the application of nitrogen fertilizer in Japan enhanced the vegetative growth of *L. japonica*, increased the biological yield of vegetative organs, and elevated the crude protein content of the leaves. However, it resulted in a decrease in both economic yield and chlorogenic acid content of the buds of the reproductive organs [11-14]. Adding P could not only promote flower bud differentiation and increase economic yield, but also increase the Chlorogenic acid content of flower buds and improve commodity quality [15-17]. When the recommended dosages of N, P_2O_5 and K_2O were 34.9 - 47.9, 13.1 - 14.4, 18.5 -23.8 g/plant, the economic yield of *L. japonica* was 387.0 - 430.3 CNY/plant [19].

Tongwei County locates in the hilly and gully region of the Loess Plateau in central Gansu Province. The landform in the territory is composed of hilly and gully like hills, hilly and gully, residual tableland and Sichuan tableland. The mountain ridges are undulating, and the ravines are crisscrossing[20]. The annual

sunshine duration are 1400 h, and the annual total solar radiation is 5446.5 MJ.m^{-2}. There are abundant light and heat resources, which are conducive to the development of modern Silk Road agricultural practices in cold and arid areas. Currently, a high-end *L. japonica* planting base spanning 6,667 ha has been established within the Tongwei Qingliang Yuan Limited Liability Company. This endeavor is part of the Poverty Alleviation and Development initiative in the *L. japonica* Industry. Moreover, the produced *L. japonica* has achieved a remarkable status, both in terms of quality and industry reputation, being at the forefront within China. However, due to long-term planting, there has been a significant increase in fertilizer usage for per tree, which has become a major hindrance to the development of local industries. There is an urgent need to investigate the optimal dosage of N, P, and K fertilizers for *L. japonica*. Therefore, this study conducted an experiment using "3414" Fertilizer Design experiment to establish a model and finally determine a optimal formula for cultivating *L. japonica* with high yield and economic profit.

Materials and Methods

Overview of the test site

The experiment began in April 2022. The experimental site located in Wangjiahe Community, Yaochuan Village, Lidian Township, Tongwei County (1860 m above sea level, 35 ° 25 ′ 40 ″ N, 105 ° 10 ′ 30 ″ E). It belongs to temperate semi-arid Continental climate type, with four distinct seasons, drought and little rain, and abundant light resources. The average annual temperature was 8.2℃, and the annual precipitation was 300 mm, most of them are from July to September. The frost free period was 145 d. The crops mature once a year and belong to the hilly and gully agricultural area of the Loess Plateau in central Gansu, The soil is loam. The irrigation only depended on rainfall, the water, light and other natural factors are consistent. Artificial management of weeding and uniform spraying of pesticides were used during growth period. Imidacloprid

25% wettable powder were sprayed at rate of 24 g a.i. per hectare for the plant.

The experimental site for this experiment was an authorized experimental base of Gansu Agricultural Academy, which has been approved and authorized by the provincial government to serve as an experimental field site. The development of *L. japonica* industry has unique natural advantages. The soil pH of the experimental site was 8.49. The organic matter in soil was 11.01 g/kg. total N was 0.6 g/kg, available N was 42.80 mg/kg. Total P was 0.72 g/kg, available P was 8.97 mg/kg, total K was 17.15 g/kg, and the available K was 160.67 mg/kg.

Materials

The tested *L. japonica* species was Beihua No.1 of 4 year's cultivating time. The fertilizers used in the test were mainly urea (total N content is 46%, Petro China Company Limited), granular Superphosphate (the effective P_2O_5 was 12%, Baoji Fenghe Chemical Co., Ltd.), Potassium sulfate (K_2O was 50%, Qinghai Laishuo Industry and Trade Co., Ltd.).

Method

Design of experiments

This experiment adopted quadratic regression optimal design scheme of '3414' with 3 fertilizer factors set as N, P, and K, and the levels were 0, 1, 2, and 3 [10]. There were 14 treatments totally. Among them, of which level 0 means with no fertilization; level 2 means the commonly used fertilization amount; level 1 means 1/2 of the commonly used fertilization amount; and level 3 means 3/2 of the commonly used fertilization amount. The formulized fertilizer was mixed evenly, and the fertilizer were poured into a small pit of 30 cm deep around the root of each tree. This was the first time for formula fertilization experiment carried out on the Tongwei *L. japonica* in the hilly and gully area of the Loess Plateau in central Gansu. There is no reference data for the determination of the 2 levels of each factor (the approximate value of the local optimal fertilization amount). Therefore, it was determined by reference to the local yield level in

2016-2021 and the fertilization amount in Tongwei County. The fertilization dose was 30 g N, 10 g P_2O_5, and 16 g K_2O per plant. Each treatment had 6 *L. japonica* trees, 3 replicates, arranged by random blocks, and the spacing between plants and rows was 1.0 mX1.0 m. Nitrogen fertilizer, phosphorus fertilizer, and potassium fertilizer were applied in a one-time manner on April 8, 2022 before germination. The method of fertilization was ditch application, and the fertilization amount was determined according to Table 1.

Table 1 Experimental design of ‘3414’ test.

Number	Treatment	N-coded value	P-coded value	K-coded value	Fertilization amount ($kg.hm^{-2}$)		
					N	P_2O_5	K_2O
1	$N_0P_0K_0$	0	0	0	0	0	0
2	$N_0P_2K_2$	0	2	2	0	10	16
3	$N_1P_2K_2$	1	2	2	15	10	16
4	$N_2P_0K_2$	2	0	2	30	0	16
5	$N_2P_1K_2$	2	1	2	30	5	16
6	$N_2P_2K_2$	2	2	2	30	10	16
7	$N_2P_3K_2$	2	3	2	30	15	16
8	$N_2P_2K_0$	2	2	0	30	10	0
9	$N_2P_2K_1$	2	2	1	30	10	8
10	$N_2P_2K_3$	2	2	3	30	10	24
11	$N_3P_2K_2$	3	2	2	45	10	16
12	$N_1P_1K_2$	1	1	2	15	5	16
13	$N_1P_2K_1$	1	2	1	15	10	8
14	$N_2P_1K_1$	2	1	1	30	5	8

Measurement items and methods

Yield measurement: the total yield was picked by hand and was calculated in June. The yield measurement was through the manual picking of workers, each person picked six trees, the flower weight of each tree was weighed with a

high-precision electronic balance (model AE001, Jiangsu Hongli weighing Equipment Co., LTD.), and the yield of each tree was recorded. The average value of the flower yield of six trees was the yield of a single tree.

Soil total nitrogen content was determined by NY/T 53-1987 standard Kjeldt nitrogen determination method, soil total phosphorus content was determined by NY/T 88-1988 standard molybdenum-antimony resistance colorimetric method, and soil total potassium content was determined by NY/T 87-1988 standard acid melting flame photometer and FP6450 type flame photometer.

Data analysis

DPS7.5 software and Exce1 2010 software were used to fit the univariate quadratic, binary quadratic and ternary quadratic fertilizer effect functions of yield and fertilizer application, and whether they comply with typical fertilizer efficiency models was analyzed. If it belongs to an atypical fertilizer efficiency model, the frequency analysis method was used to calculate the economic optimal fertilizer amount and the maximum yield fertilizer amount.

The frequency analysis calculation formula was [13, 20]:

$$\overline{x_i} = \frac{1}{n_j}\sum_{j=0}^{p} u_j\, n_{ij} (i = 1,2,3) \qquad (1)$$

In the formula, n_{ij} represents the number of occurrences of the variable value X_{ij}, where (u_j), and n_j represents the total number of occurrences of the variable X_i.

Standard deviation:

$$S\overline{x_i} = \frac{1}{\sqrt{n_j-1}} \cdot \sqrt{\sum_{j=0}^{p}\left(u_j - \overline{x_i}\right)^{\cdot 2} n_{ij}} \qquad (2)$$

Mean standard deviation:

$$S_x = \frac{S}{\sqrt{n_j}} \qquad (3)$$

95% confidence interval：

$$\left(\overline{x_i} - S\overline{x_i} \cdot t_{0.05(n_j-1)}\right) \sim \left(\overline{x_i} + S\overline{x_i} \cdot t_{0.05(n_j-1)}\right) \qquad (4)$$

The economic benefits were calculated as follows:

Output value (CNY/plant) = Yield of per plant*24/1000 (5)

Fertilizer input (CNY/plant) = (nitrogen fertilizer amount of per plant *4.8/1000) + (phosphorus fertilizer amount of per plant *6/1000) + (potassium fertilizer amount of per plant *8/1000) (6)

Yield to investment ratio (%)= output value /fertilizer input (7)

Result

N and P fertilizers promoting the increase of yield and economic benefits of *L. japonica* per plant

Soil fertility is the basic resource for sustainable agricultural development, nitrogen and phosphorus are the essential nutrients for crop growth and development, nitrogen and phosphorus affect the growth and development of crops, and then affect their yield and economic benefits [18]. Guo Yu et al.'s study on Qianhu found that reasonable distribution of soil nitrogen and phosphorus nutrients can effectively increase the accumulation of soil available nutrients and rhizosphere nutrients, and promote the increase of yield [18]. The index of soil nutrient abundance and deficiency was expressed as the percentage of yield in the nutrient deficient area to the total yield in fertilizer deficient area. That is, the relative yield can express the abundance and deficiency of soil nutrients [5]. Soil nutrients with a relative yield below 50% are extremely low; 50% -75% is low; 75% -95% is medium; greater than 95% is high. Thus, the index of soil nutrient abundance and deficiency of a certain crop in a certain area and the corresponding fertilizer amount were determined [19-20]. It is high to determine the soil nutrient abundance and deficiency index of a certain crop in a certain area and the corresponding fertilization amount. From Table 2, it can be seen that the contribution rate of basic soil fertility in the field trial site is 73.97%. The yield in the non fertilized area accounts for 73.97% of the total yield in the fully fertilized area, the yield in the N deficient area accounts for 84.67% of the total

yield in the fully fertilized area, the yield in the P deficient area accounts for 78.70% of the total yield in the fully fertilized area, and the yield in the K deficient area accounts for 80.72% of the total yield in the fully fertilized area. The findings indicated that the soil's available nitrogen, phosphorus, and potassium content fell within the moderate range, with the soil's capacity to supply these nutrients being at an intermediate level. Therefore, the P content of soil is the primary fertility factor that limits the yield of *L. japonica*. Within the recommended fertilization range, each application with 1 g of N, P, and K fertilizer can increase the yield of buds by 2.04 g, 8.52 g, and 4.82 g, respectively. The results showed that increasing P fertilizer had a significant effect on improving yield of *L. japonica* buds.

Table 2 The relative yield and increasing rate of fertilizer deficiency treatments

Processing number		Yield (g/plant)	Relative yield (%)	Increased yield of 1g fertilizer production of bud fresh weight（g）		
				N	P_2O_5	K_2O
1	$N_0P_0K_0$	295.77	73.97	-	-	-
2	$N_0P_2K_2$	338.53	84.67	2.04	-	-
4	$N_2P_0K_2$	314.67	78.70	-	8.52	-
8	$N_2P_2K_0$	322.73	80.72	-	-	4.82
6	$N_2P_2K_2$	399.83	100.00	-	-	-

From Table 3, it can be seen that the yield of *L. japonica* per plant was greatly affected by different treatments. Among them, the yield and economic benefits of $N_2P_3K_2$ treatment were the highest at 409 g per plant and 1.3499 USD per plant. The treatment of $N_2P_3K_2$ increased the yield significantly by 38.18% compared to the control, while the yield and economic benefits of the non fertilized control were the lowest on 296 g per plant and 0.9770 USD per plant. The highest output to investment ratio was the treatment of $N_1P_2K_1$, which reached 47.26. However, the lowest output ratio for output to investment ratio was $N_2P_2K_3$, which was 23.96. For treatments of $N_0P_2K_2$, $N_1P_2K_2$, $N_2P_2K_2$,

$N_3P_2K_2$, and $N_2P_0K_2$, $N_2P_1K_2$, $N_2P_2K_2$, and $N_2P_3K_2$ in Table 3,With the increase of nitrogen and phosphate fertilizer amount, the yield per plant of *L. japonica* increased gradually. From the treatments of $N_2P_2K_2$, $N_2P_2K_0$, $N_2P_2K_1$, and $N_2P_2K_3$ in From Table 2, it can be seen that as rate of P increases in production, the yield increased at low ratios and reached its highest value at treatment of No.6. However, when the amount of P application continued to increase until to treatment 10, the yield of per plant did not increase any more, but decreased slightly. The results of interaction relationship of fertilizer was PK>NP>NK, and the yield increased 14.46%, 9.12% and 6.39%, respectively compared with treatment with no fertilizer.

Table 3 Effects of different treatments on *L. japonica* yield and economic benefits

Experiment number		Yield（g/plant）	Increased profit than control (%)	Economic profit (CNY/ plant）	Fertilizer cost (CNY/ plant）	Ratio of profit to investment
1	$N_0P_0K_0$	295.77 ± 2.85j	-	7.10	0.000	-
2	$N_0P_2K_2$	338.5 ± 2.21h	14.46	8.12	0.188	43.22
3	$N_1P_2K_2$	389.7 ± 5.30c	31.76	9.35	0.260	35.97
4	$N_2P_0K_2$	314.67 ± 9.08i	6.39	7.55	0.272	27.76
5	$N_2P_1K_2$	378.5 ± 10.41de	27.97	9.08	0.302	30.08
6	$N_2P_2K_2$	399.83 ± 8.13ab	35.19	9.60	0.332	28.90
7	$N_2P_3K_2$	408.70 ± 2.86a	38.18	9.81	0.362	27.10
8	$N_2P_2K_0$	322.73 ± 1.16i	9.12	7.75	0.204	37.97
9	$N_2P_2K_1$	372.27 ± 4.92ef	25.86	8.93	0.268	33.34
10	$N_2P_2K_3$	395.27 ± 1.68bc	33.64	9.49	0.396	23.96
11	$N_3P_2K_2$	407.93 ± 4.14a	37.92	9.79	0.404	24.23
12	$N_1P_1K_2$	367.80 ± 4.78f	24.35	8.83	0.230	38.38
13	$N_1P_2K_1$	386.73 ± 5.14cd	30.76	9.28	0.196	47.36
14	$N_2P_1K_1$	353.4 ± 10.83g	19.49	8.48	0.238	35.64

Note: The '-' indicates does not exist, the unit price of *L. japonica* is 3.3024 USD/kg, N is 0.6605 USD/ kg,

P_2O_5 is 0.8256 USD/kg, and $K_2O$is 1.1008 USD/kg.

Analysis of fertilizer effect function

Analysis of Ternary Quadratic Fertilizer Effect Function

Using DPS software to perform regression analysis between independent variables x and y (yield per plant), the fertilizer effect function was calculated as: $Y=296.66-0.81X_1+11.53X_2+4.05X_3-0.051X_1^2-0.60X_2^2-0.28X_3^2+0.13X_1*X_2+0.20X_1*X_3-0.029X_2*X_3$; where 0.81, 11.53 and 4.05 are the absolute values of the first term of the equation, respectively. The results showed that the yield of *L. japonica* was affected by the single factor of N, P, and K in the order of P > K > N. Indicating that it can be seen that P fertilizer had the greatest impact on the yield of honeysuckle, while N fertilizer had the smallest effect. This result was consistent with results in Table 2.

The correlation coefficient R^2 was 0.9938 (P=0.0018<0.05) obtained by using DPS software for multiple regression analysis and test. It passed F-test and reached a significant level. It showed that there was a high correlation between independent variables x and y (yield). and there was a significant regression relationship between yield of *L. japonica* and N, P, K fertilizers, but the coefficient of the first term in the equation had both positive and negative values, which did not conform to the typical ternary quadratic fertilizer effect function. The calculation of recommended fertilization amount by using the marginal derivative method was not consistent with actual production. Therefore, frequency analysis method is used to calculate the recommended fertilization amount.

Analysis of single factor fertilizer effect function Single factor refers to fixing the fertilizer level of two factors and the relationship equation between fertilizer change and yield change was fitted. From Table 2, it can be seen that the fitting of the single factor fertilizer effect function was the same as that of the 3 factors, and the fertilizer usage and yield of N, P, and K fertilizers were applied for fitting. Regression analysis was conducted on N fertilizer effect function and correlation

coefficients with F and P values using treatments N numbered 2, 3, 6, and 11 in Table 1. Similarly, the effect equations of P fertilizer were fitted using treatments 4, 5, 6, and 7, while the effect equations of K fertilizer were fitted using treatments 6, 8, 9, and 10. The effect equations of each fertilizer are detailed in Table 2. The results showed that the correlation coefficient R of the fertilizer effect function of N fertilizer was 0.9868 ($P=0.1621>0.05$), and the regression effect of the equation was not significant. The correlation coefficient R of the fertilizer effect function of P fertilizer was 0.9955, ($P=0.0944>0.05$), and the regression effect of the equation is not significant. The correlation coefficient R of the fertilizer effect function of K fertilizer is 0.9990 ($P=0.0440<0.05$), and the regression effect of the equation is significant.

From Table 4 and Table 5, it can be seen that the maximum fertilization amounts of N, P_2O_5, and K_2O for each factor in the single factor fertilizer effect function are 7.5, 13, and 19.29, respectively. The corresponding maximum fertilization amounts are 408.45 g/ plant, 409.03 g/plant, and 445.99 g/plant, respectively.

Table 4 Analysis on single factor fertilizer effect function

Factor	Fertilizer effect function	R^2	P
N fertilizer	$Y=340.95+3.60X_1-0.048X_1^2$	0.9868	0.1621
P fertilizer	$Y=316.55+14.31X_2-0.55X_2^2$	0.9955	0.0944
K fertilizer	$Y=322.40+8.11X_3 -0.21X_3^2$	0.9990	0.0440

Table 5 Single factor fertilizer effect function each factor fertilizer application and maximum yield analysis

Factor functions	Maximum fertilization amount of each factor（g/plant）	Maximum output（g/plant）
Fertilizer effect function of N	7.5	408.45
Fertilizer effect function of P	13	409.03
Fertilizer effect function of K	19.29	445.99

Analysis of Two-Factor Fertilizer Effect Function

The two-factor fertilizer equation was an equation that fixes level of one

fertilizer unchanged and fits the changes in the levels of two fertilizers with the changes in yield. The fitting of the interaction effect function between the two factors was the same as that of the three factors. From Table 6, it can be seen that the regression analysis was performed on the fertilizer dosage and yield through NP, NK, and PK fertilizer related treatments. The interaction effect equation of NP fertilizer was obtained through regression analysis between the fertilizer dosage and yield of treatments numbered 2, 3, 4, 5, 6, 7, 11, and 12 in Table 1. Similarly, the interaction effect equation of NK fertilizer was obtained through regression analysis of fertilizer dosage 2 and yield under treatments of 2, 3, 6, 8, 9, 10, 11, and 13. The interaction effect equation of PK fertilizer was obtained through regression analysis of fertilizer dosage 2 and yield under treatments of 4, 5, 6, 7, 8, 9, 10, and 14. The results showed that the correlation coefficient R^2 of the fertilizer effect function of NP fertilizer was 0.9946, P=0.0269<0.05, indicating a significant regression effect of the equation; The correlation coefficient R^2 of the fertilizer effect function of NK fertilizer was 0.9898, P=0.0498<0.05, and the regression effect of the equation was significant; The correlation coefficient R^2 of the fertilizer effect function of PK fertilizer was 0.9948, P=0.0259<0.05, and the equation regression effect was significant.

Table 6 Analysis on fertilizer effect function of two factors

Factor	Fertilizer effect function	R^2	P
NP fertilizer	$Y=303.10+1.94X_1+9.72X_2-0.051X_1^2-0.60X_2^2+0.18X_1*X_2$	0.9946	0.0269
NK fertilizer	$Y=382.48-0.80X_1+1.35X_3-0.042X_1^2-0.24X_3^2+0.25X_1*X_3$	0.9898	0.0498
PK fertilizer	$Y=260.41+11.11X_2+7.36X_3-0.50X_2^2-0.24X_3^2+0.15X_2*X_3$	0.9948	0.0259

Production frequency analysis

According to the above analysis, the regression effects of N and P fertilizers were not significant in the single factor fertilizer effect function, while the regression effects of K fertilizers were significant. The regression effects of NP, NK, and PK fertilizers were significant in the fertilizer effect functions of the interaction

of two factors. The ternary quadratic fertilizer effect function was not a typical ternary quadratic fertilizer effect function. Therefore, this study was not suitable for using the marginal derivative method to calculate the maximum yield fertilization amount and the optimal fertilization amount. For results that did not fit the typical ternary quadratic fertilizer effect function, the yield frequency analysis method could be used to determine the appropriate recommended fertilization amount.

Setting Statistical Intervals (Yield Range)

According to the results of the field experiment (Table 7), the yield range was set. The maximum actual average yield of this experimental community was 409 g/plant, and the minimum was 296 g/plant. The yield range (statistical interval) was set to $296<y<409$. Frequency analysis was calculated based on the field yield statistical table of *L. japonica*.

Table 7 Field trial yield of *L. japonica*

Number	Treatment	Nutrient consumption (g.plant $^{-1}$)			Yield per plant (g.plant $^{-1}$)			
		N	P_2O_5	K_2O	I	II	III	Average value
1	$N_0P_0K_0$	0	0	0	297	296	295	296
2	$N_0P_2K_2$	0	10	16	337	338	340	339
3	$N_1P_2K_2$	15	10	16	386	396	387	390
4	$N_2P_0K_2$	30	0	16	313	307	325	315
5	$N_2P_1K_2$	30	5	16	376	370	390	379
6	$N_2P_2K_2$	30	10	16	391	402	407	400
7	$N_2P_3K_2$	30	15	16	406	412	408	409
8	$N_2P_2K_0$	30	10	0	324	323	322	323
9	$N_2P_2K_1$	30	10	8	370	369	378	372
10	$N_2P_2K_3$	30	10	24	395	397	394	395
11	$N_3P_2K_2$	45	10	16	407	413	405	408

12	$N_1P_1K_2$	15	5	16	364	373	366	368
13	$N_1P_2K_1$	15	10	8	390	381	390	387
14	$N_2P_1K_1$	30	5	8	359	341	360	353

Yield frequency analysis

The frequency of each factor level of nitrogen, phosphorus and potassium falling into the set yield range was counted respectively, and the average, standard deviation, and 95% confidence interval recommended fertilization amount of nitrogen, phosphorus, and potassium according to formulas (1) - (4) were calculated to obtain the yield frequency analysis of honeysuckle, as shown in Table 8. By calculation, the times of nitrogen fertilizer yield falling into the set interval at 0-3 level were 5, 9, 21 and 2 times, the times of phosphate fertilizer yield falling into the set interval at 0-3 level were 5,9,24 and 3 times, and the times of potassium fertilizer yield falling into the set interval at 0-3 level were 5,9,24 and 3 times, respectively. The average fertilization rates of nitrogen, phosphorus, and potassium fertilizers were 23.57,7.86 and 12.57, respectively. The recommended fertilization amount for nitrogen fertilizer (N) is 18.25-26.75 g/plant, for phosphorus fertilizer (P_2O_5) is 6.27-8.73 g/plant, and for potassium fertilizer (K_2O) is 10.04-13.96 g/plant, resulting in a yield of 376.45-397.94 g/plant.

Table 8 Frequency analysis of *L. japonica* output

Fertilization level	N fertilization amount			P fertilization amount			K fertilization amount		
	Fertilization amount (g/plant)	Frequency	Rate(%)	Fertilization amount (g/plant)	Frequency	Rate (%)	Fertilization amount (g/plant)	Frequency	Rate (%)
0	0	5	13.51	0	5	12.20	0	5	12.20
1	15	9	24.32	5	9	21.95	8	9	21.95
2	30	21	56.76	10	24	58.54	16	24	58.54
3	45	2	5.41	15	3	7.32	24	3	7.32
Sum of times		37			41			41	
x_i average value	22.5			7.5			12		
Standard deviation	13.17			4.05			6.40		

(s)			
Standard deviation	2.17	0.63	1.00
95%confidence interval	18.25-26.75	6.27-8.73	10.04-13.96

Calculation and Optimization of Fertilization Rate and Yield

The average application rates of nitrogen, phosphorus, and potassium fertilizers obtained by frequency analysis was 22.5 g/plant, 7.5 g/plant, and 12 g/plant, which were brought into the ternary quadratic fertilizer effect function, and the optimized fertilization yield was calculated to be 386.95 g/plant.

Discussion

Nitrogen, phosphorus, and potassium are the three primary nutrient elements critical for plant growth and development, exhibiting interactive effects within the plant body. Nitrogen plays a key role in the synthesis of proteins, nucleic acids, and chlorophyll, thereby promoting photosynthesis and enhancing both the yield and quality of crops. Phosphorus, an essential component of nucleic acids and nuclear proteins, increases the number of buds and the thousand-grain weight, ultimately contributing to improved yields in honeysuckle. Potassium is absorbed by plant roots in the form of ions and plays a crucial role in fundamental metabolic processes, thereby influencing plant growth and development as well as enhancing crop stress resistance. When nitrogen, phosphorus, and potassium fertilizers are applied to the soil, the various nutrient elements interact with soil microorganisms and organisms, promoting mutual enhancement of their effects. This interaction maximizes the efficacy of nitrogen, phosphorus, and potassium fertilizers, ultimately improving fertilizer utilization rates and enhancing both crop yield and quality [22-23].In this study, the yield of L. japonica increased with the increase of nitrogen and phosphorus content.

Inorganic elements such as nitrogen (N), phosphorus (P), and potassium (K) are the mineral elements with the highest demand for plant metabolism, tissue

and organ formation, and overall growth and development. Fertilization has been employed in agricultural production to satisfy the requirements of plants for these three essential elements [24]. Cao et al. [25] demonstrated that balanced fertilization of N, P, and K significantly enhances plant growth and development, as evidenced by increases in the number of flower nodes, fresh weight of flowers, and the number of flower buds, ultimately improving flower bud yield.Research has demonstrated that fertilization can enhance the yield of *L. japonica*. As the level of fertilization increases, the yield per plant also rises. However, when the amount of fertilization surpasses a certain threshold, the yield experiences a significant decline [26, 27].A single experiment conducted on nitrogen (N), phosphorus (P), and potassium (K) revealed that the order of their effect on increasing the yield of *L. japonica* is as follows: N > P > K. The optimal application rates for these nutrients were determined to be 0.1 kg/plant for N, 0.2 kg/plant for P, and 0.1 kg/plant for K, respectively [28].The results of the yield study indicated that nitrogen (N), phosphorus (P), potassium (K), and compound fertilizers can enhance the differentiation of flower buds in honeysuckle plants, which is positively correlated with the length of the flower buds.Among these elements, nitrogen (N) has the potential to enhance the yield of both leaves and flowers. In contrast, phosphorus (P) serves as the primary fertility factor influencing flower bud weight and yield, playing a crucial role in the growth and development of flower buds [29].In this study, varying fertilizer ratios significantly influenced the yield of *L. japonica* per plant. Specifically, the yield for treatment $N_2P_3K_2$ increased by 38.18% compared to the control. Additionally, the contribution rate of basic soil fertility at the field trial site was found to be 73.97%.Within the recommended range for fertilization, the application of 1 g of nitrogen, phosphorus, and potassium fertilizer has the potential to increase the yield of *L. japonica* buds by 2.04 g, 8.52 g, and 4.82 g, respectively.The results indicated that phosphorus (P) fertilizer was the primary limiting factor for

enhancing the yield of *L. japonica* buds. Therefore ,it is essential to consider the amount of phosphorus fertilizer applied to achieve high yields and a finding that aligns with the results of Zeng Huijie et al. [24]. Tongwei County locates in the hilly and gully region of the Loess Plateau in central Gansu, the correlation coefficient (R^2) of the equation function representing the fertilizer effect of *L. japonica* was established through regression analysis. This analysis was based on the yield of *L. japonica* obtained from experiments and the amounts of nitrogen (N), phosphorus (P), and potassium (K). The resulting R^2 value was 0.9938, with a p-value of 0.0018, which is less than 0.05, indicating a significant regression effect.However, the coefficients of the first terms were -0.81, 11.53, and 4.05, respectively. These values represent typical fertilizer effect functions and do not conform to the law of diminishing returns.The F-test yielded a p-value of 0.0018, which is less than the significance level of 0.05, indicating a statistically significant result. Additionally, the marginal analysis method was employed to determine the optimal fertilization amount and the maximum yield fertilization amount; however, these findings were inconsistent with actual production levels.The recommended fertilization amount for 4-year-old *L. japonica*, determined through frequency analysis, was as follows: nitrogen fertilizer (N) should be applied at a rate of 18.25-26.75 g per plant. The recommended amount for phosphorus fertilizer (P_2O_5) is 6.27-8.73 g per plant, while for potassium fertilizer (K_2O), the suggested amount ranges from 10.04 to 13.96 g per plant. The expected yield per plant is between 376.45 and 397.94 g.The conventional fertilization amounts, based on local yield levels and data from Tongwei County between 2016 and 2021, were determined to be 30 g/plant of pure nitrogen, 10 g/plant of P_2O_5, and 16 g/plant of K_2O. These amounts were found to be higher than the recommended fertilization levels derived from frequency analysis. Consequently, the recommended fertilization amount for the upcoming year's tests should be adjusted to a slightly lower level.

In summary, due to soil texture, and fertility, fertilization strategies should be tailored to the specific conditions of each planting region. Additionally, it is essential to ensure a balanced application of inorganic fertilizers to prevent soil compaction and imbalances in microbial flora, thereby enhancing the development of the *L. japonica* industry.

Conclusion

This study demonstrated that the application of formula fertilization with varying ratios of nitrogen (N), phosphorus (P), and potassium (K) can significantly enhance the yield of fresh buds of *L. japonica* in areas of China characterized by poor soil nutrient availability. The use of formula fertilizer containing N, P, and K improved soil nutrient levels to a medium grade, with phosphorus content identified as the primary limiting factor affecting yield.Increasing the doses of nitrogen (N), phosphorus (P), and potassium (K) fertilizers to the recommended levels resulted in a significant enhancement in yield. Consequently, the yield per plant reached 409 g, yielding an economic benefit of 1.3499 USD per gram per plant. This represents a substantial increase of 38.18% in yield, along with a notable economic benefit of 0.3729 USD per gram per plant compared to the control group. To model this effect, the following function was derived: $Y=296.66-0.81X_1+11.53X_2+4.05X_3-0.051X_1^2-0.60X_2^2-0.28X_3^2+0.13X_1\times X_2+0.20X_1 \times X_3-0.029X_2 \times X_3$. This study examines the combined application of N, P_2O_5, and K_2O fertilizers per plant. Through frequency analysis, the recommended doses of N, P_2O_5, and K_2O fertilizers were determined to be within the ranges of 18.25 to 26.75 g/plant, 6.27 to 8.73 g/plant, and 10.04 to 13.96 g/plant, respectively. The findings of this research have practical implications for the cultivation and production of *L. japonica*, offering valuable insights for optimizing cultivation practices and maximizing the potential of this important medicinal plant within the specific geographical

conditions of the cultivation area.

Funding

National key research and development project and sub-project (2021YFD1100504，2021YFD1100504-1，2021YFD1100504-2). Agricultural Science and technology independent innovation special key research and development program of Gansu Academy of Agricultural Sciences(2021GARS11). the Gansu Provincial Agricultural Science and Technology Special Project（GNKJ-2021-33）, the National Natural Science Foundation of China (32260550).The National Key Research and Development Project and its sub-projects (2021YFD1100504, 2021YFD1100504-1, 2021YFD1100504-2) are national-level scientific research projects on honeysuckle currently being carried out by the project team, These three projects have made significant contributions in terms of personnel allocation, technical support, and financial support.The Agricultural Science and technology independent innovation special key research and development program of Gansu Academy of Agricultural Sciences (2021GARS11),the Gansu Provincial Agricultural Science and Technology Special Project（GNKJ-2021-33）and the National Natural Science Foundation of China (32260550) provided Sharing of technical exchanges and experimental bases.

Conflicts of Interest

The authors have no conflicts of interest to declare.

Availability of data and materials

All datasets used and/or analyzed during the current study are available within the manuscript.

References：

1. Liu Z, Cheng Y, Chao Z. A Comprehensive Quality Analysis of Different Colors of Medicinal and Edible Honeysuckle. Foods. 2023;12(16):

3126. https://doi.org/10.3390/foods12163126

2. Xiao O, Li M, Chen D, Chen J, Simal-Gandara J, Dai X, et al. The dissipation, processing factors, metabolites, and risk assessment of pesticides in honeysuckle from field to table. Journal of Hazardous Materials. 2022;431:128519. https://pubmed.ncbi.nlm.nih.gov/35231811/

3. Qin S, Chen X, Jiang C, Li M, Yuan Y, Yang J, et al. Pruning induced yield and quality variations and the correlated gene expression and phytohormone changes in Lonicera japonica. Industrial Crops and Products. 2019;132:386-95. https://www.cabidigitallibrary.org/doi/full/10.5555/20193253630

4. Ivona E, Mirela C, Emil Ch,Madalina B,Monica S,Mihaela S.Influence of cultivar and fertilization with vermicompost on fruit quality and yield in lonicera spp. Current Trends in Natural Sciences,2021,10(19):72-79. https://doi.org/10.47068/ctns.2021.v10i19.009

5. Guo R,Liu R Y, Huang H Y, Zhai Y Sh,Chen Y,liW D. Effects of Different Fertilization Formulas on Yield and Quality of LoniceYa japonica Flos and Soil Microecology in Loess Plateau. Journal of Nuclear Agricultural Sciences.2023,37(7):1452-1461. https://doi.org/10.11869/j.issn.10008551.2023.07.1452

6. Peng X P,Zhang J Y,Su Y Y,Sun L Q,Liu X H,Tang X Q,et al. Effect s of spraying copper and borax fertilizer on growth physiology of Lo nicera japonica Thunb.and quality of Lonicera japonica flos .Journal o f Nanjing Agricultural University,2023,46(1):33-41. https://doi.org/10．7 685/jnau．202202004

7. Zhang X, Li S, An X, Song Z, Zhu Y, Tan Y, et al. Effects of nitro gen, phosphorus and potassium formula fertilization on the yield and berry quality of blueberry. Plos one. 2023;18(3):e0283137. https://doi.o rg/10.1371/journal.pone.0283137

8. Zhao F, Li F, Zhou J, Sun X, Wang Y, Jing L, et al. Soiltesting formula fertilization with organic fertilizer addition for target yield cannot stand long due to stem lodging of rice. Frontiers in Plant Science.2022;13:1091156. https://doi.org/10.3389/fpls.2022.1091156
9. Bao Q-p, Han X-r, Cui Z-g, Yin Y, Zhang H, Li W-r, et al. Recom mended fertilization model for spring maize nitrogen fertilizer in Nort heast China. Journal of Plant Nutrition and Fertilizers. 2020;26(4):705-16. https://10.11674/zwyf.19247
10. Saquee FS, Diakite S, Kavhiza NJ, Pakina E, Zargar M. The Efficacy of Micronutrient Fertilizers on the Yield Formulation and Quality of Wheat Grains. Agronomy. 2023;13(2):566. https://doi.org/10.3390/agronomy13020566
11. Guo L, Qiao J, Zhou D, Qin D, Huo J. Effect of Vermicompost on Fruit Quality, Growth, and Rhizosphere Soil Enzyme Activities of Blue Honeysuckle (Lonicera caerulea L.). Journal of Soil Science and Plant Nutrition. 2023:1-9. https://springer.longhoe.net/article/10.1007/s42729-023-01300-9
12. Guo R, Liu R Y, Huang H Y, Zhai Y Sh, Chen Y, Li W D. Effects of Different Fertilization Formulas on Yield and Quality of Lonicer a japonica Flos and Soil Microecology in Loess Plateau. Journal of N uclear Agricultural Sciences,2023,37(7):1452~1461. https://doi10.11869/j. issn.1000-8551.2023.07.1452.
13. Chen Y, Zhou X, Ma L, Lin Y, Huang X. Chinese yam yield is affected by soil nutrient levels and interactions among N, P, and K fertilizers. Chinese Herbal Medicines. 2023. https:// pii/S1674638423000448
14. Liao G, Wang Y, Yu H, He P, Lin Z, Dai T, et al. Nutrient use effic iency has decreased in southwest China since 2009 with increasing ris k of nutrient excess. Communications Earth & Environment. 2023;4(1):

388. https://www.nature.com/articles/s43247-023-01036-5

15. Iseki K, Ikazaki K, Batieno BJ. Heterogeneity effects of plant density and fertilizer application on cowpea grain yield in soil types with different physicochemical characteristics. Field Crops Research. 2023;292:108825. https://doi.org/10.1016/j.fcr.2023.108825

16. Yang L, Lin Y, Kong J, Yu Y, He Q, Su Y, et al. Effects of fertilization and dry-season irrigation on the timber production and carbon storage in subtropical Eucalyptus plantations. Industrial Crops and Products. 2023;192:116143. https://doi.org/10.1016/j.indcrop.2022.116143

17. Tang S, Zhou J, Pan W, Sun T, Liu M, Tang R, et al. Effects of combined application of nitrogen, phosphorus, and potassium fertilizers on tea (Camellia sinensis) growth and fungal community. Applied Soil Ecology. 2023;181:104661. https://doi.org/10.1016/j.apsoil.2022.104661

18. Guo Y, Xu G, Luo Sh, Luo M,Tan Q Sh,Yang Y D, et al.Effects of nitr ogen,phosphorus and potassium mixed fertilizer application on yield and quality dynamic of *peuce-danum praeruptorum* Dunn. Chinese soil andf ertilizer,2023,(10):169-176.https://doi.org/10.11838/sfsc.1673-6257.22539.

19. Fontes PCR, Braun H, Busato C, Cecon PR. Economic optimum nitro gen fertilization rates and nitrogen fertilization rate effects on tuber ch aracteristics of potato cultivars. Potato Research. 2010;53:167-79. https: //springer.longhoe.net/article/10.1007/s11540-010-9160-3

20. Li H, Zhang Y, Sun Y, Liu P, Zhang Q, Wang X, et al. Long-term effects of optimized fertilization, tillage and crop rotation on soil fertility, crop yield and economic profit on the Loess Plateau. European Journal of Agronomy. 2023;143:126731. https://doi.org/10.1016/j.eja.2022.126731

21. Niu Sh W,Wang Zh F, Li G Zh, Ma L B. Analyzing the Potentials and Structure of Rural Household Energy Use in Hilly Regions of the Loess Plateau：A Case Study in Qizuei Village of Tongwei County o

f Gansu Province. Resources Science. 2007,29(3):105-110. https://doi.org/1007-7588(2007)03-0105-06

22. Wang Q, Wang X-c, Li X-y, Wang B, Gao X-q, Fu B-z. Effect of “3414” Fertilization on Seed Yield and Quality of Bromus inermis in Arid Area of Ningxia. Acta Agrestia Sinica. 2022,30(12):3470. http://doi.org/10.48130/grares-0024-0007

23. XIONG Y, LIU H CH, WANG YP, PAN F G. Effects of Nitrogen, Phosphorus, and Potassium Dosage and Ratio on Seed Yield and Quality of "Qianhuo No. 1”[J] .Journal of Shandong Agricultural University(Natural Science Edition), 2024,55(1):009-015. https://www.cnki.net/KCMS

24. Zeng H, Wang X, Qiao Z, Li Y, Cai N, Liu S, et al. Influence on yield and quality of Lonicera japonica by soil testing and formulated fertilization. J Nucl Agric Sci. 2017;31:2443-9.

25. Cao Y, Pan Y, Wang M, Liu T, Meng X, Guo S. The effects of diff erent nitrogen forms on chlorophyll fluorescence and photosystem II i n Lonicera japonica. Journal of Plant Growth Regulation. 2023;42(7):4 106-17. https://springer.longhoe.net/article/10.1007/s00344-022-10873-1

26. Gardner Z. Effects of Fertilization and Drying Conditions on the Quality of Selected Chinese Medicinal Plants. 2019. https://orcid.org/0000-0001-5351-5276

27. Chen K, Ma L, Chen C, Liu N, Wang B, Bao Y, et al. Long-Term Impact of N, P, K Fertilizers in Different Rates on Yield and Quality of Anisodus tanguticus (Maxinowicz) Pascher. Plants. 2023;12(11):2102.

28. Jourdan C, Silva EV, Gonçalves JLM, Ranger J, Moreira RM, Laclau JP. Fine root production and turnover in Brazilian Eucalyptus plantations under contrasting nitrogen fertilization regimes. Forest ecology and management. 2008;256(3):396-404. https://link.springer.com/article/10.1007/s11368-013-0718-y

29. Zeng P, Guo Z, Xiao X, Peng C, Liu L, Yan D, et al. Physiological stress responses, mineral element uptake and phytoremediation potential of Morus alba L. in cadmium-contaminated soil. Ecotoxicology and environmental safety. 2020;189:109973. https://doi: 10.1016/j.ecoenv.2019.109973

Response to Reviewer Comments

Dear Editors and Reviewers:

We are very grateful for the further comments concerning our manuscript entitled **"Study on Nitrogen, Phosphorus and Potassium Formula Fertilization Model of Lonicera japonica Thunb in hilly and gully Loess Plateau of China (PONE-D-24-06358)".** These comments are all valuable and very helpful for the further revision and improvement of our article, which also provides an important guideline for our researches and scientific writing. We have carefully studied the comments and made corrections that we hope meet the standards for publication. If you have any questions regarding this manuscript, please do not hesitate to contact me. The detailed corrections in the article and the responses to the editors' and reviewers' comments are as follows:

Response to Editor Requirements:

1.Line 40. Please Change CNY by dollars or euros? Frequently, all international journal use USA dollars.

Answer: Based on Exchange rate between US Dollar and RMB on December 12, 2024:1USD≈7.2674CNY　1CNY≈ 0.1376USD

The yuan in the original text is completely revised to US dollars.(Line40)

2.Line 47. Remove Lonicera genus in the

Answer: “belongs to　Lonicera genus in the *Caprifoliaceae* family” instead “belongs to　the Caprifoliaceae family” (Line46-47)

3.Line 100. Please put soil texture. This information is essential to exchange cationic capacity.

Answer: The soil is loam。(Line101)

4.Line 103 is it grams of active ingredient? If yes. It should be g ai.Please consider this sentence:　Imidacloprid wettable (25%), powder with 1000 fold solution, were spray at rate of 24 g per hectare for the plant.

Answer: yes.　“Imidacloprid wettable (25%), powder with 1000 fold solution, were spray at rate of 24 g per hectare for the plant” instead “ Imidacloprid 25% wettable powder were sprayed at rate of 24 g a.i. per hectare for the plant” (line103-104)

5.108. Please put space in　L. japonica

Line 113. Why do the authors not mention K in the title? And line 198 mention K

Answer: The original has been amended.(line108)

The author mentions potassium in the title.

6.122. I think 3 fertilizers and 4 levels involve 40 treatments. Why the research was done with only 14 treatments.

$N_0Q_0P_0$; $N_0Q_0P_1$; $N_0Q_1P_1$; $N_0Q_1P_2$; $N_0Q_2P_2$; $N_0Q_2P_3$; $N_0Q_3P_3$; $N_0Q_1P_3$; $N_0Q_2P_3$; $N_0Q_3P_2$; $N_1Q_0P_0$; $N_1Q_0P_1$; $N_1Q_1P_1$; $N_1Q_1P_2$; $N_1Q_2P_2$; $N_1Q_2P_3$; $N_1Q_3P_3$; $N_1Q_1P_3$; $N_1Q_2P_3$; $N_1Q_3P_2$; $N_2Q_0P_0$; $N_2Q_0P_1$; $N_2Q_1P_1$; $N_2Q_1P_2$; $N_2Q_2P_2$; $N_2Q_2P_3$; $N_2Q_3P_3$; $N_2Q_1P_3$; $N_2Q_2P_3$; $N_2Q_3P_2$; $N_3Q_0P_0$; $N_3Q_0P_1$; $N_3Q_1P_1$; $N_3Q_1P_2$; $N_3Q_2P_2$; $N_3Q_2P_3$; $N_3Q_3P_3$; $N_3Q_1P_3$; $N_3Q_2P_3$;$N_3Q_3P_2$.

Answer: 3414 test design 14 processing is because it is a special design based on Latin square design, this design can effectively control the test error and improve the accuracy of the test.

7. Line 173 and 174. Under this title explain the amount of N, P and K o the soil as a result; however, there is not information in material of methods about how to do this research

Answer: Soil total nitrogen content was determined by NY/T 53-1987 standard Kjeldt nitrogen determination method, soil total phosphorus content was determined by NY/T 88-1988 standard molybdenum-antimony resistance colorimetric method, and soil total potassium content was determined by NY/T 87-1988 standard acid melting flame photometer and FP6450 type flame photometer.(line147-152)

8. Line 216 and 217 both cite that fertilizer increase the yield per plant

Answer: “it can be seen that N and P fertilizers can promote the increase of yield per plant ,and the yield per plant of L. japonica gradually increases with the increase of fertilizer amount. ”instead“With the increase of nitrogen and phosphate fertilizer amount, the yield per plant of L. japonica increased gradually. ”(line220-222)

9.The authors should check the references. For example: Number 23 is not cited in the text.

Answer: The original has been amended.(line357)

10.Line 319. Delete one standard deviation

Answer: Deleted from the original text.(line322)

11.Line from 340 to 349 is information according to introduction section no discussion section.

Answer: Thank you for your valuable suggestions！ We have corrected the manuscript as you required. “In this study, the yield of L. japonica increased with the increase of nitrogen and phosphorus content.”(line357-359)

12.Line 339 please put L. Japonica in cursive in the discussion and conclusion section.

Answer: The original has been amended.(line368,373,388)

13.Line 388-390. The two sentence join by a comma has not sense together.

Answer: Thank you for your valuable suggestions！ We have

corrected the manuscript as you required. “The results indicated that phosphorus (P) fertilizer was the primary limiting factor for enhancing the yield of L. japonica buds. Therefore, it is essential to consider the amount of phosphorus fertilizer applied to achieve high yields and a finding that aligns with the results of Zeng Huijie et al.” (line388-392)

14.Line 413: in summery…. has information that not was previously mentioned such as weather variation or texture.

Answer: Thank you for your valuable suggestions！ We have corrected the manuscript as you required. “In summary, due to soil texture, and fertility, fertilization strategies should be tailored to the specific conditions of each planting region. Additionally, it is essential to ensure a balanced application of inorganic fertilizers to prevent soil compaction and imbalances in microbial flora, thereby enhancing the development of the L. japonica industry.”(line418-422)
