# Supplementary material for: Nitrogen, phosphorus and potassium formula fertilization model of Lonicera japonica Thunb in hilly and gully Loess Plateau of China
Source: PLoS One. 2025 Jun 4;20(6):e0323828. doi: 10.1371/journal.pone.0323828 (PMC12136625; doi:10.1371/journal.pone.0323828)
Supplement: Attachment — Submitted filename: Plos One 18,3,2024.docx [file pone.0323828.s002.docx]

The study presented in this paper investigates the effects of various fertilization treatments on the yield and economic benefits of Lonicera japonica (L. japonica). The authors conducted a comprehensive field trial, analyzed the relative yield and increasing rate of fertilizer deficiency treatments, performed regression analysis for fertilizer effect functions, and conducted yield frequency analysis to determine the optimal fertilization rates for maximizing yield.

The paper begins by introducing the concept of soil nutrient abundance and deficiency index, which is crucial for understanding the impact of different fertilization treatments on crop yield. The authors categorize soil nutrients based on their relative yield percentages and establish a correlation between soil fertility and the yield of L. japonica. Notably, the results highlight phosphorus (P) as the primary fertility factor limiting the yield of L. japonica, indicating the importance of balanced fertilization strategies. The paper also delves into the analysis of fertilizer effect functions, including ternary quadratic and single-factor functions. Through regression analysis and correlation coefficients, the authors identify the relative impact of N, P, and K fertilizers on L. japonica yield. The study concludes with yield frequency analysis, which calculates recommended fertilization amounts based on statistical intervals, optimizing fertilization rates for maximizing yield.

Overall, the paper presents a well-structured and scientifically rigorous analysis of fertilization treatments' effects on L. japonica yield and economic benefits. The findings contribute significantly to agricultural practices, particularly in optimizing fertilization strategies for enhancing crop productivity and profitability.

**Abstract**

The abstract provides a clear overview of the study's objectives and methods. However, there are a few grammatical issues that need to be addressed, such as:

"Formula fertilization is effective in increasing yield and economic profit in medicinal plants, but no study on performance on Lonicera japonica Thunb ( L. japonica) has been conducted, which is an important medicinal plant." - This sentence is a bit convoluted. It could be rephrased for better clarity.

"The results showed the application of N, P and K content in soil improved soil nutrient effectively." - It would be helpful to specify how the soil nutrient content was improved by the application of N, P, and K fertilizers.

**Introduction**

The introduction provides a good background on Lonicera japonica and its medicinal properties. However, some areas require further explanation or elaboration:

The statement "Formula fertilization is an effective way to improve yield and quality of L. japonica" could be supported with references or previous studies demonstrating this effectiveness.

It would be beneficial to include more details about the formula fertilization trial mentioned in the introduction, such as the specific dosages and ratios of N, P, and K used.

The paragraph discussing the geographical conditions and cultivation practices in Tongwei County could be expanded to include information on climate, soil types, and any unique challenges faced in cultivating L. japonica.

Overall, the abstract and introduction provide a solid foundation for the study, but addressing these points would enhance the clarity and comprehensiveness of the paper.

**Result**

**Table 2** provides a detailed overview of the relative yield and increasing rate of fertilizer deficiency treatments, demonstrating the incremental yield associated with nitrogen (N), phosphorus (P2O5), and potassium (K2O) fertilizers. The data suggest that increasing P fertilizer has a significant positive effect on improving the yield of L. japonica buds, aligning with the earlier observation regarding the importance of P in soil fertility.

1. Can you provide more details about the methodology used to determine the relative yield and increasing rate of fertilizer deficiency treatments presented in Table 2?
2. How were the yield values in Table 2 calculated, particularly for the different fertilization treatments (N0P0K0, N0P2K2, N2P0K2, etc.)?

**Table 3** showcases the effects of different treatments on L. japonica yield and economic benefits. The authors discuss the yield-per-plant variations across various fertilization treatments, emphasizing the substantial increase in yield and economic profit with optimized fertilization strategies. The analysis of output-to-investment ratios provides valuable insights into the cost-effectiveness of different fertilization regimes, with treatments like N1P2K1 exhibiting a notably high output ratio.

1. What specific factors were considered when analyzing the economic benefits in Table 3? Did the analysis include factors such as market prices, production costs, or other economic variables?
2. Can you elaborate on the criteria used to determine the highest output-to-investment ratio in Table 3? How does this ratio contribute to the economic assessment of different fertilization treatments?
3. The results in Table 3 show a significant increase in yield and economic profit with certain fertilization treatments. Could you discuss any potential limitations or challenges associated with implementing these treatments on a larger scale or in different agricultural contexts?
4. How were the fertilization amounts for nitrogen (N), phosphorus (P2O5), and potassium (K2O) determined in Table 3? Were these amounts based on theoretical calculations, experimental data, or existing guidelines?

**Discussion**

The discussion effectively highlights the importance of N, P, and K fertilization in promoting plant growth and development. However, it would be beneficial to provide more specific details on how these elements interact and contribute to yield improvement in Lonicera japonica. For example, what mechanisms are involved in promoting flower bud differentiation and yield increase with N, P, and K fertilizers?

- The discussion mentions the impact of different fertilizer ratios on the yield of L. japonica and presents recommended fertilization amounts. It's important to elaborate on how these ratios and amounts were determined. Was there a specific methodology or experimental design used to establish these recommendations?
- Additionally, it would be helpful to discuss any potential limitations or challenges in implementing these recommended fertilization amounts, especially considering variations in soil conditions and environmental factors across different planting regions.

The discussion briefly compares the findings of this study with previous research, which is good for contextualizing the results. However, it could be expanded by discussing any discrepancies or similarities in results between this study and others in the field.

Overall, the discussion provides a thorough analysis of the results but could benefit from further elaboration on the methodology, comparative analysis with previous studies, and practical recommendations for growers and researchers.
