# Supplementary material for: Nitrogen, phosphorus and potassium formula fertilization model of Lonicera japonica Thunb in hilly and gully Loess Plateau of China
Source: PLoS One. 2025 Jun 4;20(6):e0323828. doi: 10.1371/journal.pone.0323828 (PMC12136625; doi:10.1371/journal.pone.0323828)
Supplement: Attachment — Submitted filename: Remarks to the Author and editor.docx [file pone.0323828.s003.docx]

Remarks to the Author and editor

Manuscript Number PONE-D-24-06358

Title: Study on Nitrogen, Phosphorus and Potassium Formula Fertilization Model of Lonicera japonica Thunb in hilly and gully Loess Plateau of China

General comments

The present study was conducted to the aim of this study is to establish models for higher yield of L. japonica by formula fertilization with different ratios of nitrogen (N), phosphorus (P) and potassium (K) in main producing areas in hilly and gully Loess Plateau of China. A ‘3414’ formula fertilization trial with different dose and ratios of N, P, and K used for L. japonica was investigated. The results showed the application of N, P and K content in soil improved soil nutrient effectively. And P was identified as the primary limiting factor for yield. The ratio of output to investment of N2P2K3 was lowered significantly. The fertilizer effect function was: Y = 296.66 - 0.81X1 + 11.53X2 + 4.05X3 - 0.051X12 - 0.60X22 - 0.28X32 + 0.13X1×X2 + 0.20X1×X3 - 0.029X2×X3. The recommended dose of N, P, and K fertilizer was 18.25-26.75 g, 6.27-8.73 g, and 10.04-13.96 g, respectively, per plant. By applying the fertilizer with formula from this research, the yield and economic benefit increased notably by 38.18% and 2.71 CNY.

The objectives of the study are clear, methodology is understandable, and discussion is well written, apart from that, there are a few suggestions for the improvement of this paper, which are as followed

1. In abstracts some figurative result outcomes should be further elaborated.
2. The number of keywords should be at least five.
3. Gansu Province is a main producing area in China, of what? please clarify the statement.
4. At Line number 70, s should be small in Sunshine.
5. Use proper language, at line number 86 replace the word “test site” with experimental site and at 87 test began, as experiment began.
6. Avoid unnecessary capitalization of words for example at line number 89 “Continental” The Times at line number 281 and 283.
7. In material and method section, please describe the soil preparation for cultivation of L. japonica species, irrigation and agronomic practices adopted for this experiment.
8. Line number 126 Yield measurement, which yield parameters were recorded, please elaborate.
9. Line number 153, Do not use bracket in the beginning of the sentence as you did (It is high to determine the soil nutrient abundance and deficiency index of a certain crop in a certain area and the corresponding fertilization amount.).
10. Line number 163, potassium is on an average degrade? Please check this sentence.
11. How economic benefit was calculated please describe a formula or method in material & method section.
12. Increase number of references, add them at appropriate places.
