# Supplementary material for: Nitrogen, phosphorus and potassium formula fertilization model of Lonicera japonica Thunb in hilly and gully Loess Plateau of China
Source: PLoS One. 2025 Jun 4;20(6):e0323828. doi: 10.1371/journal.pone.0323828 (PMC12136625; doi:10.1371/journal.pone.0323828)
Supplement: Attachment — Submitted filename: RevisionPONE-D-24-06358R4.docx [file pone.0323828.s007.docx]

The manuscript has a clear objective, with appropriate statistical analyses and acceptable writing quality. However, some data acquisition methods or calculations described in the Results section would be more suitable in the Materials and Methods section. The obtained results are clear and practically applicable for producers. The optimization of fertilizer use, a key point of this manuscript, leads to higher economic yield. Additional considerations are noted below to clarify or enhance its presentation. The manuscript is recommended for acceptance with minor formal revisions.

- Line 40. Please Change CNY by dollars or euros? Frequently, all international journal use USA dollars.
- Line 47. Remove Lonicera genus in the
- Line 100. Please put soil texture. This information is essential to exchange cationic capacity.
- Line 103 is it grams of active ingredient? If yes. It should be g ai.

Please consider this sentence: Imidacloprid wettable (25%), powder with 1000 fold solution, were spray at rate of 24 g per hectare for the plant.

- 108. Please put space in *L. japonica*

Line 113*.* Why do the authors not mention K in the title? And line 198 mention K

- 122. I think 3 fertilizers and 4 levels involve 40 treatments. Why the research was done with only 14 treatments.
- N_0_Q_0_P_0_ ; N_0_Q_0_P_1_; N_0_Q_1_P_1_ ; N_0_Q_1_P_2_ ; N_0_Q_2_P_2_ ; N_0_Q_2_P_3_ ; N_0_Q_3_P_3_ ; N_0_Q_1_P_3_; N_0_Q_2_P_3_; N_0_Q_3_P_2_ ; N_1_Q_0_P_0_ ; N_1_Q_0_P_1_; N_1_Q_1_P_1_ ; N_1_Q_1_P_2_ ; N_1_Q_2_P_2_ ; N_1_Q_2_P_3_ ; N_1_Q_3_P_3_ ; N_1_Q_1_P_3_; N_1_Q_2_P_3_; N_1_Q_3_P_2_ ; N_2_Q_0_P_0_ ; N_2_Q_0_P_1_; N_2_Q_1_P_1_ ; N_2_Q_1_P_2_ ; N_2_Q_2_P_2_ ; N_2_Q_2_P_3_ ; N_2_Q_3_P_3_ ; N_2_Q_1_P_3_; N_2_Q_2_P_3_; N_2_Q_3_P_2_ ; N_3_Q_0_P_0_ ; N_3_Q_0_P_1_; N_3_Q_1_P_1_ ; N_3_Q_1_P_2_ ; N_3_Q_2_P_2_ ; N_3_Q_2_P_3_ ; N_3_Q_3_P_3_ ; N_3_Q_1_P_3_; N_3_Q_2_P_3_;N_3_Q_3_P_2_.

* Line 173 and 174. Under this title explain the amount of N, P and K o the soil as a result; however, there is not information in material of methods about how to do this research

*Line 216 and 217 both cite that fertilizer increase the yield per plant

- The authors should check the references. For example: Number 23 is not cited in the text.
- Line 319. Delete one standard deviation
- Line from 340 to 349 is information according to introduction section no discussion section.
- Line 339 please put L. Japonica in cursive in the discussion and conclusion section.
- Line 388-390. The two sentence join by a comma has not sense together.
- Line 413: in summery…. has information that not was previously mentioned such as weather variation or texture.
