# Supplementary material for: Nitrogen, phosphorus and potassium formula fertilization model of Lonicera japonica Thunb in hilly and gully Loess Plateau of China
Source: PLoS One. 2025 Jun 4;20(6):e0323828. doi: 10.1371/journal.pone.0323828 (PMC12136625; doi:10.1371/journal.pone.0323828)
Supplement: Attachment — Submitted filename: Response to Reviewer Comments.doc [file pone.0323828.s004.doc]

**Response to Reviewer Comments**

**Dear Editors and** **Reviewers:**

We are very grateful for the further comments concerning our manuscript entitled **“Study on Nitrogen, Phosphorus and Potassium Formula Fertilization Model of　Lonicera japonica Thunb in hilly and gully Loess Plateau of China (PONE-D-24-06358)”.** These comments are all valuable and very helpful for the further revision and improvement of our article, which also provides an important guideline for our researches and scientific writing. We have carefully studied the comments and made corrections that we hope meet the standards for publication. If you have any questions regarding this manuscript, please do not hesitate to contact me. The detailed corrections in the article and the responses to the editors' and reviewers' comments are as follows:

**Response to Editor Requirements:**

“When submitting your revision, we need you to address these additional requirements.”

1. Please ensure that your manuscript meets PLOS ONE's style requirements, including those for file naming. The PLOS ONE style templates can be found at <https://journals.plos.org/plosone/s/file?id=wjVg/PLOSOne_formatting_sample_main_body.pdf> and <https://journals.plos.org/plosone/s/file?id=ba62/PLOSOne_formatting_sample_title_authors_affiliations.pdf>

**Answer:** The paper has been modified according to The PLOS ONE style templates.

2. In your Methods section, please provide additional information regarding the permits you obtained for the work. Please ensure you have included the full name of the authority that approved the field site access and, if no permits were required, a brief statement explaining why.

**Answer:** The experimental site for this experiment was an authorized experimental base of Gansu Agricultural Academy, which has been approved and authorized by the provincial government to serve as an experimental field site. And we have corrected as you required. (Line 100-102)

3. Thank you for stating the following financial disclosure: "National key research and development project and sub-project (2021YFD1100504，2021YFD1100504-1，2021YFD1100504-2). Agricultural Science and technology independent innovation special key research and development program of Gansu Academy of Agricultural Sciences(2021GARS11); Gansu Province Science and Technology Plan project: Livelihood Science and technology special - Topics on rural revitalization(22CX2NA007); National Natural Science Foundation of China (NSFC)(31460547)" Please state what role the funders took in the study.  If the funders had no role, please state: "The funders had no role in study design, data collection and analysis, decision to publish, or preparation of the manuscript." If this statement is not correct you must amend it as needed. Please include this amended Role of Funder statement in your cover letter; we will change the online submission form on your behalf.

**Answer:** The National Key Research and Development Project and its sub-projects (2021YFD1100504, 2021YFD1100504-1, 2021YFD1100504-2) are national-level scientific research projects on honeysuckle currently being carried out by the project team. These three projects have made significant contributions in terms of personnel allocation, technical support, and financial support.The Agricultural Science and technology independent innovation special key research and development program of Gansu Academy of Agricultural Sciences (2021GARS11) provided Sharing of technical exchanges and experimental bases. (Line 409-416)

4. Thank you for stating the following in your Competing Interests section:  
"no" Please complete your Competing Interests on the online submission form to state any Competing Interests. If you have no competing interests, please state "The authors have declared that no competing interests exist.", as detailed online in our guide for authors at http://journals.plos.org/plosone/s/submit-now

**Answer:** The authors have declared that no competing interests exist, and have changed the online submission form as you required.

5. PLOS requires an ORCID iD for the corresponding author in Editorial Manager on papers submitted after December 6th, 2016. Please ensure that you have an ORCID iD and that it is validated in Editorial Manager. To do this, go to ‘Update my Information’ (in the upper left-hand corner of the main menu), and click on the Fetch/Validate link next to the ORCID field. This will take you to the ORCID site and allow you to create a new iD or authenticate a pre-existing iD in Editorial Manager. Please see the following video for instructions on linking an ORCID iD to your Editorial Manager account: <https://www.youtube.com/watch?v=_xcclfuvtxQ>

**Answer:** The authors have registered an account as you required.

**Response to the Reviewer#１**

1. The abstract section does not provide an overview of the research methods

**Answer:** we have modified as required. (Line 29-30)

2. The number of keywords should be at least five.

**Answer:** we have modified as required.

3. Gansu Province is a main producing area in China, of what? please clarify the statement.

**Answer:** Gansu Province is the main producing area of most authentic Chinese medicinal materials in China. (Line 46-47)

4. At Line number 70, s should be small in Sunshine.

**Answer:** we have modified as required. (Line 72)

5. Use proper language, at line number 86 replace the word “test site” with experimental site and at 87 test began, as experiment began.

**Answer:** we have modified as required. (Line89)

6. Avoid unnecessary capitalization of words for example at line number 89 “Continental” The Times at line number 281 and 283.

**Answer:** we have modified as required. (Line 306-308)

7. In material and method section, please describe the soil preparation for cultivation of L. japonica species, irrigation and agronomic practices adopted for this experiment.

**Answer:** we have modified as required. The experiment located in Tongwei, Gansu Province, the temperate belongs to semi-humid and semi-arid monsoon climate type. Therefore, the irrigation only depended on rainfall, the water, light and other natural factors are consistent. Artificial management of weeding and uniform spraying of pesticides were used during growth period. 24g of 25% imidacloprid wettable powder with 1000 fold solution were spray per hectare for the plant. (Line 96-99).

The formulized fertilizer was mixed evenly, and the fertilizer were poured into a small pit of 30 cm deep around the root of each tree. (Line 120-121).

8. Line number 126 Yield measurement, which yield parameters were recorded, please elaborate.

**Answer:** we have modified as required. The yield measurement was through the manual picking of workers, each person picked six trees, the flower weight of each tree was weighed with a high-precision electronic balance (model AE001, Jiangsu Hongli weighing Equipment Co., LTD.), and the yield of each tree was recorded. The average value of the flower yield of six trees was the yield of a single tree. (Line 137-141)

9. Line number 153, Do not use bracket in the beginning of the sentence as you did (It is high to determine the soil nutrient abundance and deficiency index of a certain crop in a certain area and the corresponding fertilization amount.)

**Answer:** we have modified as required. (Line 174-176)

10. Line number 163, potassium is on an average degrade? Please check this sentence.

**Answer:** we have modified as required. (Line182-184)

11. How economic benefit was calculated please describe a formula or method in material & method section.

**Answer:** we have described the information as you required. (Line159-164)

The economic benefits were calculated as follows:

Output value (CNY/plant) = Yield of per plant*24/1000	(5)

Fertilizer input (CNY/plant) = (nitrogen fertilizer amount of per plant *4.8/1000) + (phosphorus fertilizer amount of per plant *6/1000) + (potassium fertilizer amount of per plant *8/1000)

Yield to investment ratio (%) = output value /fertilizer input (7)

12. Increase number of references, add them at appropriate places.

**Answer:** we have corrected as you required.

**Response to the Reviewer#２**

**1.** The abstract provides a clear overview of the study's objectives and methods. However, there are a few grammatical issues that need to be addressed, such as:

"Formula fertilization is effective in increasing yield and economic profit in medicinal plants, but no study on performance on Lonicera japonica Thunb ( L. japonica) has been conducted, which is an important medicinal plant." - This sentence is a bit convoluted. It could be rephrased for better clarity.

"The results showed the application of N, P and K content in soil improved soil nutrient effectively." - It would be helpful to specify how the soil nutrient content was improved by the application of N, P, and K fertilizers.

**Answer:** we have corrected as you required. (Line 23-24, 32)

**2.** The introduction provides a good background on Lonicera japonica and its medicinal properties. However, some areas require further explanation or elaboration. The statement "Formula fertilization is an effective way to improve yield and quality of *L. japonica*" could be supported with references or previous studies demonstrating this effectiveness. It would be beneficial to include more details about the formula fertilization trial mentioned in the introduction, such as the specific dosages and ratios of N, P, and K used. The paragraph discussing the geographical conditions and cultivation practices in Tongwei County could be expanded to include information on climate, soil types, and any unique challenges faced in cultivating *L. japonica*. Overall, the abstract and introduction provide a solid foundation for the study, but addressing these points would enhance the clarity and comprehensiveness of the paper.

**Answer:** we have corrected as you required. (Line 53,58,96-99)

**3.** Table 2 provides a detailed overview of the relative yield and increasing rate of fertilizer deficiency treatments, demonstrating the incremental yield associated with nitrogen (N), phosphorus (P_2_O_5_), and potassium (K_2_O) fertilizers. The data suggest that increasing P fertilizer has a significant positive effect on improving the yield of *L. japonica* buds, aligning with the earlier observation regarding the importance of P in soil fertility. Can you provide more details about the methodology used to determine the relative yield and increasing rate of fertilizer deficiency treatments presented in Table 2? How were the yield values in Table 2 calculated, particularly for the different fertilization treatments (N0P0K0, N0P2K2, N2P0K2, etc.)?

**Answer:** The yield measurement was through the manual picking of workers, each person picked six trees, the flower weight of each tree was weighed with a high-precision electronic balance. The yield of each tree was recorded. The average value of the flower yield of six trees was the yield of a single tree. (Line 136-141).

**4.** Table 3 showcases the effects of different treatments on *L. japonica* yield and economic benefits. The authors discuss the yield-per-plant variations across various fertilization treatments, emphasizing the substantial increase in yield and economic profit with optimized fertilization strategies. The analysis of output-to-investment ratios provides valuable insights into the cost-effectiveness of different fertilization regimes, with treatments like N_1_P_2_K_1_ exhibiting a notably high output ratio. What specific factors were considered when analyzing the economic benefits in Table 3? Did the analysis include factors such as market prices, production costs, or other economic variables? Can you elaborate on the criteria used to determine the highest output-to-investment ratio in Table 3? How does this ratio contribute to the economic assessment of different fertilization treatments?

**Answer:**

The frequency analysis calculation formula was:

 (1)

In the formula, n_ij_ represents the number of occurrences of the variable value X_ij_, where (u_j_), and n_j_ represents the total number of occurrences of the variable X_i_.

Standard deviation:

 (2)

Mean standard deviation:

 (3)

95% confidence interval：

 (4)

The economic benefits were calculated as follows:

**Output value** (CNY/plant) = Yield of per plant*24/1000	(5)

Fertilizer input (CNY/plant) = (nitrogen fertilizer amount of per plant *4.8/1000) + (phosphorus fertilizer amount of per plant *6/1000) + (potassium fertilizer amount of per plant *8/1000)	(6)

**Yield to investment ratio** (%)= output value /fertilizer input	(7)

5. The discussion effectively highlights the importance of N, P, and K fertilization in promoting plant growth and development. However, it would be beneficial to provide more specific details on how these elements interact and contribute to yield improvement in *Lonicera japonica*. For example, what mechanisms are involved in promoting flower bud differentiation and yield increase with N, P, and K fertilizers? 	The discussion mentions the impact of different fertilizer ratios on the yield of L. japonica and presents recommended fertilization amounts. It's important to elaborate on how these ratios and amounts were determined. Was there a specific methodology or experimental design used to establish these recommendations?	Additionally, it would be helpful to discuss any potential limitations or challenges in implementing these recommended fertilization amounts, especially considering variations in soil conditions and environmental factors across different planting regions. The discussion briefly compares the findings of this study with previous research, which is good for contextualizing the results. However, it could be expanded by discussing any discrepancies or similarities in results between this study and others in the field.

**Answer:** Thank you for your valuable suggestions, we have corrected as you required. (Line 322-334)
