# Supplementary material for: Nitrogen, phosphorus and potassium formula fertilization model of Lonicera japonica Thunb in hilly and gully Loess Plateau of China
Source: PLoS One. 2025 Jun 4;20(6):e0323828. doi: 10.1371/journal.pone.0323828 (PMC12136625; doi:10.1371/journal.pone.0323828)
Supplement: Attachment — Submitted filename: Yang--Response to Reviewer Comments.docx [file pone.0323828.s006.docx]

**Response to Reviewer Comments**

**Dear Editors and** **Reviewers:**

We are very grateful for the further comments concerning our manuscript entitled **“Study on Nitrogen, Phosphorus and Potassium Formula Fertilization Model of　Lonicera japonica Thunb in hilly and gully Loess Plateau of China (PONE-D-24-06358)”.** These comments are all valuable and very helpful for the further revision and improvement of our article, which also provides an important guideline for our researches and scientific writing. We have carefully studied the comments and made corrections that we hope meet the standards for publication. If you have any questions regarding this manuscript, please do not hesitate to contact me. The detailed corrections in the article and the responses to the editors' and reviewers' comments are as follows:

**Response to Editor Requirements:**

1. **Journal Requirements:**
   Please review your reference list to ensure that it is complete and correct. If you have cited papers that have been retracted, please include the rationale for doing so in the manuscript text, or remove these references and replace them with relevant current references. Any changes to the reference list should be mentioned in the rebuttal letter that accompanies your revised manuscript. If you need to cite a retracted article, indicate the article’s retracted status in the References list and also include a citation and full reference for the retraction notice.
    **Answer:** I have carefully reviewed my paper references and they are complete and there are no references to papers that have been retracted.
2. **Reviewer #4:** The manuscript is well-prepared and I can see that the authors responded to some comments, as this is my first time to review this manuscript, I have noticed that the results section need to be revised. only present the results with no explanation or interpretation or comparison with previous work.
   These can be done in the discussion section.

**Answer:** The discussion section has been modified in the original text according to the reviewer's suggestion.

**3. Reviewer #5:** The authors have well addressed the comments made previously. I recommend it accepting for publication after revising some minor corrections. In line (62-66) Could you please revise this long sentence into two clear sentences? This will make it easier to understand by clearly distinguishing between the different effects of nitrogen fertilizer.
Please follow the author guidelines for standard formatting of the manuscript.

**Answer:**  Thank you for your valuable suggestions！ We have corrected the manuscript as you required. “The application of nitrogen fertilizer in Japan enhanced the vegetative growth of L. japonica, increased the biological yield of vegetative organs, and elevated the crude protein content of the leaves. However, it resulted in a decrease in both the economic yield and the chlorogenic acid content of the buds of the reproductive organs. ”(Line62-67)
