# Supplementary material for: Nitrogen, phosphorus and potassium formula fertilization model of Lonicera japonica Thunb in hilly and gully Loess Plateau of China
Source: PLoS One. 2025 Jun 4;20(6):e0323828. doi: 10.1371/journal.pone.0323828 (PMC12136625; doi:10.1371/journal.pone.0323828)
Supplement: Attachment — Submitted filename: Reviewer Comments PONE-D-24-06358_R4.docx [file pone.0323828.s009.docx]

**Reviewer Comments:**

The manuscript entitled “**Study on Nitrogen, Phosphorus and Potassium Formula Fertilization Model of *Lonicera japonica* *Thunb* in hilly and gully Loess Plateau of China**” where the authors studied the optimizing fertilizer application *for Lonicera japonica* in the Loess Plateau of China, suffers from several significant shortcomings despite the relevance of the topic. I have some suggestions that authors should consider before the final publication of the manuscript. Therefore, the paper needs a major revision. My further comments are included below.

1. Abstract needs to be completely redrafted. This section contains numerous grammatical and punctuation errors. It would be great to concise the content for abstract section and emphasize more on your key findings, significance, and novelty of your work.
2. The manuscript contains numerous grammatical and punctuation errors line 60, 70, 74, 75, 141, 207, 247, 326, 327, 329, 330, 366, 370, 374, 377, 381, 389, 398, 410, and 429, awkward phrasing, and inconsistencies in terminology. Unit style is not uniform as line 77 and 110, Please ensure the same style throughout manuscript.
3. The description of the "3414" experiment is vague. It's crucial to clearly define the treatment levels for each factor (N, P, K) and the rationale behind their selection.
4. The method of "pouring into a small pit of 30 cm deep" lacks detail. How was the fertilizer distributed within the pit? Was any soil mixed with the fertilizer?
5. The soil analysis is limited. Information on soil pH, organic matter, and potentially other relevant parameters is missing.
6. The use of frequency analysis to determine optimal fertilization rates appears unconventional and requires thorough justification.
7. The statistical significance of the fertilizer effect function and individual treatment effects need to be more rigorously assessed.
8. The manuscript lacks a clear statement of the statistical assumptions and methods used.
9. The presentation of results is disorganized and difficult to follow.
10. The use of numerous tables and sub-sections makes the manuscript cumbersome.
11. Visual aids, such as graphs and figures, would significantly improve the clarity and readability.
12. The English language and scientific writing style require significant improvement.
13. The paragraph starting pattern was inconsistent, differing from that in lines 93 and 113, 301, 320, 337. Please maintain a consistent paragraph starting pattern throughout the manuscript. In reference section , scientific name is not italic line 487, 493, and so on.
14. The conclusions should focus on the work´s contribution and not be a summary of results.
15. The study focuses on a single location and a specific cultivar of *L. japonica*. The generalizability of the findings to other regions and cultivars is questionable. The manuscript requires substantial revision to address the shortcomings outlined above.
