# Supplementary material for: Nitrogen, phosphorus and potassium formula fertilization model of Lonicera japonica Thunb in hilly and gully Loess Plateau of China
Source: PLoS One. 2025 Jun 4;20(6):e0323828. doi: 10.1371/journal.pone.0323828 (PMC12136625; doi:10.1371/journal.pone.0323828)
Supplement: Attachment — Submitted filename: Reviewer Comments PONE-D-24-06358ding.docx [file pone.0323828.s011.docx]

**Response to Reviewer Comments**

**Dear Editors and** **Reviewers:**

We are very grateful for the further comments concerning our manuscript entitled **“Study on Nitrogen, Phosphorus and Potassium Formula Fertilization Model of　Lonicera japonica Thunb in hilly and gully Loess Plateau of China (PONE-D-24-06358)”.** These comments are all valuable and very helpful for the further revision and improvement of our article, which also provides an important guideline for our researches and scientific writing. We have carefully studied the comments and made corrections that we hope meet the standards for publication. If you have any questions regarding this manuscript, please do not hesitate to contact me. The detailed corrections in the article and the responses to the editors' and reviewers' comments are as follows:

**Response to Editor Requirements:**

1.Line 40. Please Change CNY by dollars or euros? Frequently, all international journal use USA dollars.

**Answer:** Based on Exchange rate between US Dollar and RMB on December 12, 2024:1USD≈7.2674CNY 1CNY≈ 0.1376USD

The yuan in the original text is completely revised to US dollars.(Line40)

2.Line 47. Remove Lonicera genus in the

**Answer:**“belongs to Lonicera genus in the *Caprifoliaceae* family” instead“belongs to the Caprifoliaceae family”(Line46-47)

3.Line 100. Please put soil texture. This information is essential to exchange cationic capacity.

**Answer:** The soil is loam。(Line101)

4.Line 103 is it grams of active ingredient? If yes. It should be g ai.Please consider this sentence: Imidacloprid wettable (25%), powder with 1000 fold solution, were spray at rate of 24 g per hectare for the plant.

**Answer:** yes. “Imidacloprid wettable (25%), powder with 1000 fold solution, were spray at rate of 24 g per hectare for the plant”instead“ Imidacloprid 25% wettable powder were sprayed at rate of 24 g a.i. per hectare for the plant”(line103-104)

5.108. Please put space in L. japonica

Line 113. Why do the authors not mention K in the title? And line 198 mention K

**Answer:** The original has been amended.(line108)

The author mentions potassium in the title.

6.122. I think 3 fertilizers and 4 levels involve 40 treatments. Why the research was done with only 14 treatments.

N_0_Q_0_P_0_ ; N_0_Q_0_P_1_; N_0_Q_1_P_1_ ; N_0_Q_1_P_2_ ; N_0_Q_2_P_2_ ; N_0_Q_2_P_3_ ; N_0_Q_3_P_3_ ; N_0_Q_1_P_3_; N_0_Q_2_P_3_; N_0_Q_3_P_2_ ; N_1_Q_0_P_0_ ; N_1_Q_0_P_1_; N_1_Q_1_P_1_ ; N_1_Q_1_P_2_ ; N_1_Q_2_P_2_ ; N_1_Q_2_P_3_ ; N_1_Q_3_P_3_ ; N_1_Q_1_P_3_; N_1_Q_2_P_3_; N_1_Q_3_P_2_ ; N_2_Q_0_P_0_ ; N_2_Q_0_P_1_; N_2_Q_1_P_1_ ; N_2_Q_1_P_2_ ; N_2_Q_2_P_2_ ; N_2_Q_2_P_3_ ; N_2_Q_3_P_3_ ; N_2_Q_1_P_3_; N_2_Q_2_P_3_; N_2_Q_3_P_2_ ; N_3_Q_0_P_0_ ; N_3_Q_0_P_1_; N_3_Q_1_P_1_ ; N_3_Q_1_P_2_ ; N_3_Q_2_P_2_ ; N_3_Q_2_P_3_ ; N_3_Q_3_P_3_ ; N_3_Q_1_P_3_; N_3_Q_2_P_3_;N_3_Q_3_P_2_.

**Answer:** 3414 test design 14 processing is because it is a special design based on Latin square design, this design can effectively control the test error and improve the accuracy of the test.

7. Line 173 and 174. Under this title explain the amount of N, P and K o the soil as a result; however, there is not information in material of methods about how to do this research

**Answer:** Soil total nitrogen content was determined by NY/T 53-1987 standard Kjeldt nitrogen determination method, soil total phosphorus content was determined by NY/T 88-1988 standard molybdenum-antimony resistance colorimetric method, and soil total potassium content was determined by NY/T 87-1988 standard acid melting flame photometer and FP6450 type flame photometer.(line147-152)

8. Line 216 and 217 both cite that fertilizer increase the yield per plant

**Answer:** “it can be seen that N and P fertilizers can promote the increase of yield per plant ,and the yield per plant of L. japonica gradually increases with the increase of fertilizer amount. ”instead“With the increase of nitrogen and phosphate fertilizer amount, the yield per plant of L. japonica increased gradually. ”(line220-222)

9.The authors should check the references. For example: Number 23 is not cited in the text.

**Answer:**The original has been amended.(line357)

10.Line 319. Delete one standard deviation

**Answer:** Deleted from the original text.(line322)

11.Line from 340 to 349 is information according to introduction section no discussion section.

**Answer:** Thank you for your valuable suggestions！ We have corrected the manuscript as you required. “In this study, the yield of L. japonica increased with the increase of nitrogen and phosphorus content.”(line357-359)

12.Line 339 please put L. Japonica in cursive in the discussion and conclusion section.

**Answer:** The original has been amended.(line368,373,388)

13.Line 388-390. The two sentence join by a comma has not sense together.

**Answer:** Thank you for your valuable suggestions！ We have corrected the manuscript as you required. “The results indicated that phosphorus (P) fertilizer was the primary limiting factor for enhancing the yield of L. japonica buds. Therefore, it is essential to consider the amount of phosphorus fertilizer applied to achieve high yields and a finding that aligns with the results of Zeng Huijie et al.”(line388-392)

14.Line 413: in summery…. has information that not was previously mentioned such as weather variation or texture.

**Answer:** Thank you for your valuable suggestions！ We have corrected the manuscript as you required. “In summary, due to soil texture, and fertility, fertilization strategies should be tailored to the specific conditions of each planting region. Additionally, it is essential to ensure a balanced application of inorganic fertilizers to prevent soil compaction and imbalances in microbial flora, thereby enhancing the development of the L. japonica industry.”(line418-422)
